# Chemical and Biological Characterization of the Ethyl Acetate Fraction from the Red Sea Marine Sponge *Hymedesmia* sp.

**DOI:** 10.3390/ph17060724

**Published:** 2024-06-03

**Authors:** Zeinab I. El Sayed, Wafaa H. B. Hassan, Mahmoud M. Abdel-Aal, Shaza M. Al-Massarani, Wael M. Abdel-Mageed, Omer A. Basudan, Mehtab Parveen, Eman Abdelsalam, Sahar Abdelaziz

**Affiliations:** 1 Department of Pharmacognosy, Faculty of Pharmacy, Zagazig University, Zagazig 44519, Egypt; zinab_elsayed@su.edu.eg (Z.I.E.S.); wafaahbh@zu.edu.eg (W.H.B.H.); mahmoud.ibrahim@su.edu.eg (M.M.A.-A.); eaamer@zu.edu.eg (E.A.); 2 Department of Pharmacognosy, College of Pharmacy, King Saud University, P.O. Box 2457, Riyadh 11451, Saudi Arabia; salmassarani@ksu.edu.sa (S.M.A.-M.); basudan@ksu.edu.sa (O.A.B.); 3 Department of Chemistry, Faculty of Science, Aligarh Muslim University, Aligarh 202002, India; mehtab.organic2009@gmail.com

**Keywords:** UPLC-ESI-MS/MS, Red Sea, marine sponge, *Hymedesmia* sp., secondary metabolites, antimicrobial, antioxidant, cytotoxicity

## Abstract

*Hymedesmiidae* is one of the largest families of marine sponges and stands out as an exceptional source of variable metabolites with diverse biological activities. In this study, the ethyl acetate fraction (HE) of a *Hymedesmia* sp. marine sponge from the Red Sea, Egypt, was analyzed for the first time using Ultra-performance liquid chromatography electrospray ionization tandem mass spectrometry (UPLC-ESI-MS/MS) analysis. The analysis tentatively identified 29 compounds in this fraction, including the isolation and identification of six compounds (two pyrimidine nucleosides, one purine, and two pyrimidine bases in addition to one cerebroside) for the first time. The structures of the isolated compounds were established by 1D and 2D NMR (nuclear magnetic resonance), MS (mass spectrometry), and IR (infrared) spectroscopy. Furthermore, the cytotoxic, antioxidant, and antimicrobial activities of the ethyl acetate fraction were evaluated in vitro. The fraction exhibited strong DPPH scavenging activity with an IC_50_ of 78.7 µg/mL, compared to ascorbic acid as a positive control with an IC_50_ of 10.6 µg/mL. It also demonstrated significant cytotoxic activity with IC_50_ values of 13.5 µg/mL and 25.3 µg/mL against HCT-116 and HEP-2 cell lines, respectively, compared to vinblastine as a positive control with IC_50_ values of 2.34 µg/mL and 6.61 µg/mL against HCT-116 and HEP-2, respectively. Additionally, the ethyl acetate fraction displayed promising antibacterial activity against *S. aureus* with a MIC value of 62.5 µg/mL, compared to ciprofloxacin as a positive control with MIC values of 1.56 µg/mL for Gram-positive bacteria and 3.125 µg/mL for Gram-negative bacteria. It also exhibited activity against *E. coli* and *P. aeruginosa* with MIC values of 250 µg/mL and 500 µg/mL, respectively. Briefly, this is the first report on the biological activities and secondary metabolite content of the ethyl acetate fraction of *Hymedesmia* sp. marine sponge, emphasizing the potential for further research against resistant bacterial and fungal strains, as well as different cancer cell lines. The ethyl acetate fraction of *Hymedesmia* sp. is a promising source of safe and unique natural drugs with potential therapeutic and pharmaceutical benefits.

## 1. Introduction

Marine sponges are an incredibly rich source of natural products for drug discovery. Approximately 30% of all naturally occurring metabolites discovered to date from the marine environment are derived from sponges including various classes with varying skeletons such as glycosides, sterols, peroxides, terpenes, polyketides, phenols, nucleosides, amino acid analogues, cyclic peptides, and fatty acid derivatives [1,2]. Sponges, particularly those of the phylum Porifera, are the oldest living metazoans. They have adapted so well to the drastic shifts in their environment that they were able to survive [3]. The harsh conditions of the marine environment, such as darkness, salinity, and high pressure, combined with the need to protect themselves from pathogens and predators, have led to the presence of variable and unique secondary metabolites in sponges. Due to the diversity of currently available marine natural products (MNPs), many studies have documented diverse biological activities for these compounds, including immunosuppressive, neuroprotective, anticancer, antimicrobial, and anti-inflammatory properties. Moreover, marine sponges are opening new avenues in the control of infectious diseases, as pathogens are constantly changing and developing resistance to existing drugs [4].

*Hymedesmia* sp., a marine sponge collected from the Red Sea, Egypt (20 km away from Sharm El-Sheikh [27°45′57.8″ N 34°22′10.8″ E]), belongs to the family *Hymedesmiidae*, which is one of the largest marine sponge families. It contains the largest number of species within the class *Demospongiae*. The family includes ten recognized genera, most of which have not received much attention in terms of their chemical and biological investigation, such as *Acanthancora*, *Pseudohalichondria*, *Plocamionida*, *Spanioplon*, *Myxodoryx*, and *Hymedesmia*. Various classes of biologically active secondary metabolites have been isolated from this family, including steroids, alkaloids, diterpenes, macrolides, and sesterterpenes. The isolated compounds have shown promising biological activities, such as antimicrobial, cytotoxic, anti-inflammatory, antioxidant, and hypoglycemic effects [5].

According to the available literature, nothing has been reported about *Hymedesmia* sp., a marine sponge collected from the Red Sea, Egypt, other than the study in which endozoic fungi derived from this sponge were isolated [6]. This encouraged us to carry out the present work to characterize, for the first time, the chemical constituents of the ethyl acetate fraction of *Hymedesmia* sp. marine sponge using UPLC-ESI-MS/MS analysis, the isolation and identification of the main components of this sponge, and the subsequent evaluation of its biological activities.

## 2. Results and Discussion

### 2.1. Characterization of the Isolated Compounds

**Figure 1:**
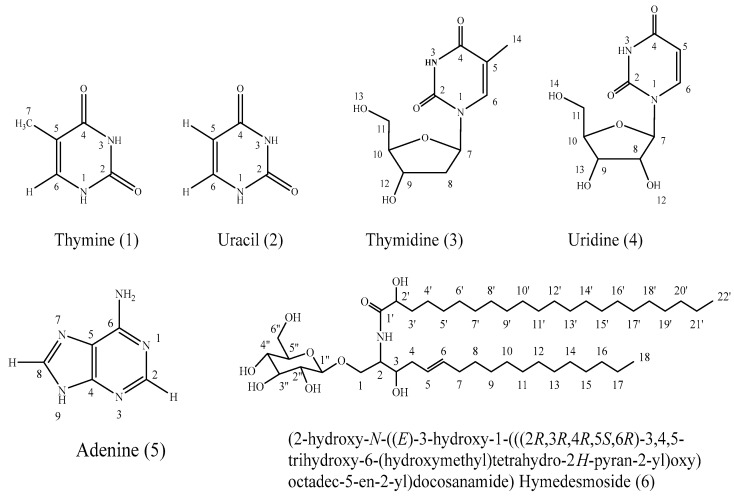
The chemical structures of compounds isolated from *Hymedesmia* sp. marine sponge.

A chemical investigation of the ethyl acetate fraction of the Red Sea marine sponge *Hymedesmia* sp. revealed the isolation of six compounds. Various spectral analyses such as UV, ESI-MS/MS, ^1^H-NMR, ^13^C-NMR, HSQC, and HMBC were used for the structure elucidation of the isolated compounds, in addition to comparison with the available literature. They were characterized as thymine (**1**) [7], uracil (**2**) [7], thymidine and uridine (**3** and **4**) [8,9], adenine (**5**) [10,11,12], and hymedesmoside (**6**) [13]. Figure 1 shows the chemical structure of the isolated compounds. To the best of our knowledge, this is the first report on the isolation of these compounds from *Hymedesmia* sp.

Compound **1** was isolated as a white crystalline powder with a melting point of 315 °C and was soluble in methanol. It was identified as thymine based on the comparison of its ESI-MS/MS (Figure S1), IR (Figure S2), ^1^H-NMR, and ^13^C-NMR (Table S1) (Figures S3 and S4) with the reported literature [7,14,15].

Compound **2** was isolated as a crystalline white powder with a melting point of 335 °C and was soluble in methanol. It was identified as uracil based on the comparison of its ESI-MS/MS (Figure S5), IR (Figure S6), ^1^H-NMR, ^13^C-NMR (Table S2), and (Figures S8 and S9) HSQC and HMBC (Figures S9 and S10) with the reported data [7,14,15].

Compounds **3** and **4** were isolated as white clusters soluble in methanol and were identified as a mixture of thymidine (major 81%) and uridine (minor 6%) based on a comparison of their spectroscopic analysis data, including EI-MS and EI-MS/MS chromatograms (Figures S11–S14), IR (Figure S15), ^1^H-NMR, and ^13^C-NMR (Table S3), and (Figures S16 and S17) HSQC and HMBC (Figures S18 and S19) with the reported data [8,9].

Compound **5** was isolated as a crystalline yellowish-white powder with a melting point of 315 °C and was soluble in methanol. It was identified as adenine based on the comparison of its EI-MS/MS (Figure S20), IR (Figure S21), and ^1^H-NMR (Figure S22) with the reported data [10,11,12].

Compound **6** was isolated as a greasy off-white powder with a melting point of 186 °C and was soluble in a dichloromethane/methanol mixture. It was identified as 2-hydroxy-*N*-((*E*)-3-hydroxy-1-[(2*R*,3*R*,4*S*,5*S*,6*R*)-3,4,5-trihydroxy-6-(hydroxymethyl) tetrahydro-2*H*-pyran-2-yl)oxy)octadec-5-en-2-yl] docosanamide (Hymedesmoside) based on the comparison of its EI-MS/MS (Figure S24), IR (Figure S23) ^1^H-NMR (Figure S25), and ^13^C-NMR (Figures S26 and S27) with the reported data [13].

### 2.2. Tentative Identification of Constituents of Hymedesmia sp. Ethyl Acetate Fraction by UPLC-ESI-MS/MS

Marine sponges are primitive multicellular invertebrates that are part of the marine biomass. They are present all over the planet and live in many ecosystems. Sponges produce a wide variety of secondary metabolites. In the current study, UPLC- ESI-MS/MS in the positive ion mode was used to analyze the *Hymedesmia* sp. ethyl acetate (HE) fraction for the first time. Compound identification was based on their MS^2^ data, including the precursor ion mass, fragments, neutral mass loss, and comparison with the available literature.

#### Characterization of the Components of *Hymedesmia sp.* Ethyl Acetate Fraction 

**Figure 2:**
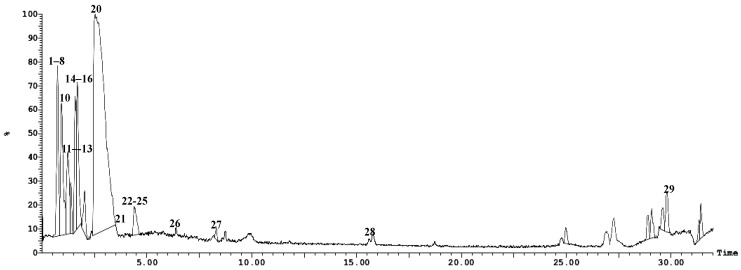
UPLC-ESI-MS chromatogram of *Hymedesmia* sp. ethyl acetate fraction in positive (+) ionization mode.

**Figure 3:**
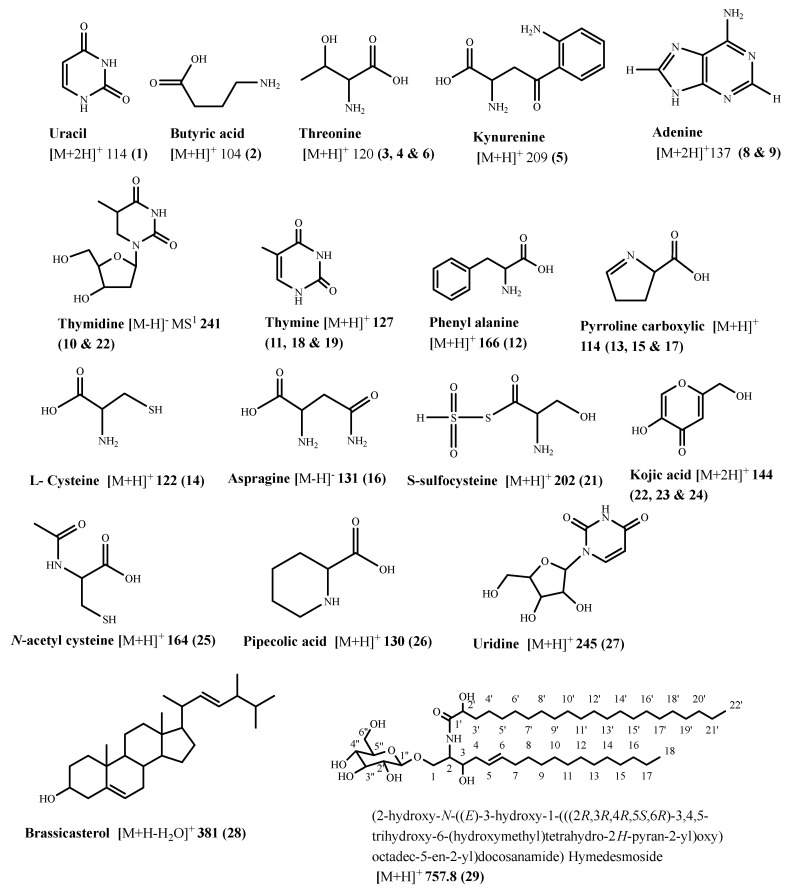
Chemical structures of the tentatively identified compounds from *Hymedesmia* sp. ethyl acetate fraction.

UPLC-ESI-MS/MS analysis of the *Hymedesmia* sp. ethyl acetate fraction led to the tentative identification of twenty-nine compounds as shown in Figure 2 and Figure 3 and Table 1. These compounds include amino acids, sterols, and cerebrosides.

Compounds **1** and **7** (R_t_. 0.21, 0.27 min) were tentatively assigned as uracil. They exhibited deprotonated and protonated molecular ion peaks [M+2H]^+^ at *m*/*z* 114 and 113. The most prominent base peak at *m*/*z* 70 [M+H-NHCO] corresponded to the loss of the carbonyl and NH groups. Based on this fragmentation pattern and comparison with literature, compounds **1** and **7** were concluded to be uracil [16] and were isolated in a purified form.

Compound **2** (R_t_. 0.74 min) ESI-MS/MS showed the molecular ion peak at *m*/*z* 104 [M+H]^+^ and MS^2^ fragment ion at *m*/*z* 59, which was produced by the loss of a carboxyl group. The daughter ion at *m*/*z* 44 corresponded to C_2_H_4_NH_2._ In addition, the fragment ion at *m*/*z* 43 showed the subsequent loss of an amino group from *m*/*z* 59. Based on comparison with the literature, compound **2** was identified as aminobutyric acid [16].

Compounds **3**, **4**, and **6** (R_t_. 0.68, 0.78, 1.88) exhibited the same molecular ion peak at *m*/*z* 120 [M+H]^+^. The MS^2^ fragmentation of these compounds showed the loss of a water molecule leaving a fragment ion at *m*/*z* 102. The loss of C_3_H_8_ON was evidenced from the fragment ion at *m*/*z* 74. Furthermore, the base peak at *m*/*z* 70 showed the loss of a methyl and 2 hydroxyl groups. From this fragmentation pattern and through comparison with the available literature [17,18], compounds **3**, **4**, and **6** were tentatively identified as threonine and its isomers.

Compound **5** (R_t_. 0.78 min) was tentatively identified as kynurenine as it revealed a protonated molecular ion peak at *m*/*z* 209 [M+H]^+^, and ESI-MS^2^ fragments at *m*/*z* 164 and 148 showed the loss of COOH and NH_2_ groups, respectively. The base peak fragment ion at *m*/*z* 136 showed the loss of C_2_H_3_NO_2_ followed by the loss of one water molecule to produce a fragment ion at *m*/*z* 118. This fragmentation style is in good agreement with that of kynurenine. It was reported that this compound protects the retina from oxidative stress and decreases the chromatic distortion, leading to a sharper retinal image [16,19].

**Figure 4:**
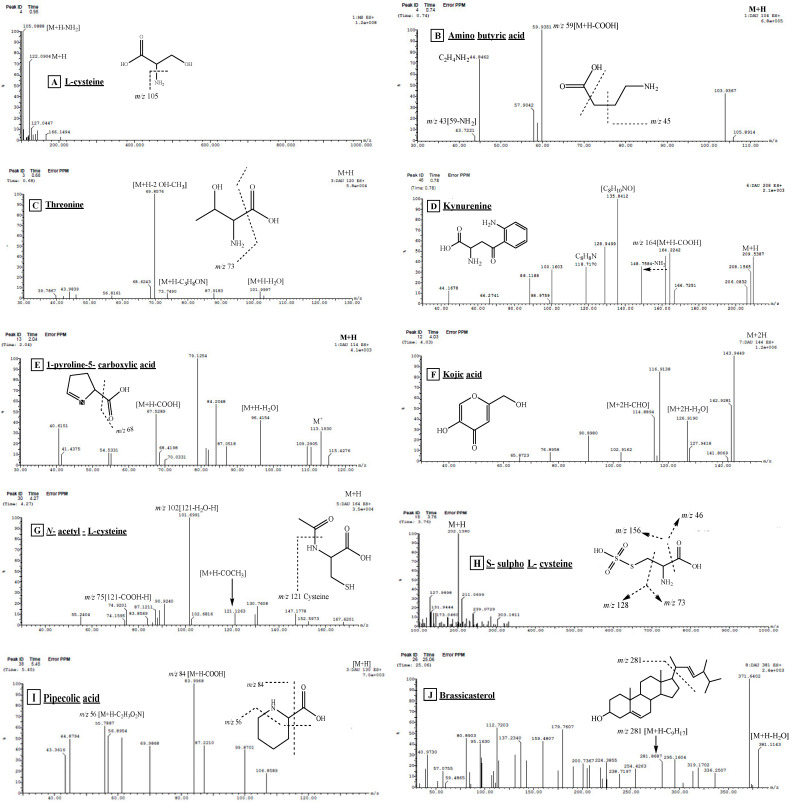
UPLC-ESI-MS/MS chromatograms of some identified compounds in ethyl acetate fraction of *Hymedesmia* sp. (HE) in positive (+) ionization mode.

Compound **25** (R_t_. 12.97 min) was tentatively identified as kynurenine with its isomers as the ESI/MS^1^ shows a protonated molecular ion peak at *m*/*z* 209 [M+H]^+^, and ESI-MS^2^ fragment ions at *m*/*z* 164 and 148 showed the loss of COOH and NH_2_ groups, respectively. The base peak fragment ion at *m*/*z* 136 showed the loss of C_2_H_3_NO_2_, followed by the loss of one molecule of water to give a fragment ion at *m*/*z* 118. This fragmentation pattern, along with other fragments shown in Figure 4D was in good agreement with that of kynurenine.

Compounds **8** and **9** (R_t_ 0.80, 0.94 min) ESI-MS/MS showed a pseudo-molecular ion peak at *m*/*z* 137 [M+2H]^+^ which was compatible with the molecular formula C_5_H_5_N_5_ [7]. The presence of adenine was supported by the remaining ESI/MS/MS fragments at *m*/*z* 119 [M^+^+H-NH_3_], 109 [M^+^+H-CHN], 93 [M^+^+H-CH_3_N_2_], and 66 [C_3_H_2_N_2_], which were in good agreement with those of adenine [20]. This compound was isolated in this work.

Compounds **10** and **20** (R_t_ 1.06, 3.48 min) were tentatively identified as thymidine and its isomers. The ESI-MS/MS in negative and positive ionization modes showed molecular ion peaks at *m*/*z* 241 and 243 corresponding to [M-H]^−^ and [M+H]^+^, respectively. The fragment ion at *m*/*z* 125 corresponds to thymine after the loss of deoxyribose sugar. Therefore, compounds **10** and **20** were identified as thymidine through comparison with the available literature [21]. The compound was isolated in this work.

Compounds **11**, **18**, and **19** (R_t_, 1.18, 2.06 and 2.08 min) were tentatively identified as thymine and its isomers based on the ESI-MS/MS spectrum, which showed a protonated molecular ion peak at *m*/*z* 127 [M+H]^+^. The MS^2^ fragment ion at *m*/*z* 84 [M+H-NHC=O] was observed, and the MS^2^ fragment at *m*/*z* 110 resulted from the elimination of the NH_3_ group. Additionally, a fragment ion at *m*/*z* 56 resulted from the loss of the carbonyl group, and another fragment ion at *m*/*z* 44 corresponded to the amide group. These data are in good agreement with the literature [22]. These compounds were isolated in this work.

Compound **12** (R_t_. 1.21 min) was tentatively identified as phenyl alanine. It exhibited ESI-MS^1^ at *m*/*z* 166 [M+H]^+^. The ESI/MS^2^ spectrum also showed a base peak fragment ion at *m*/*z* 120, indicating the loss of the COOH group. These data are in good agreement with the available literature [16].

Compounds **13**, **15**, and **17** (R_t_. 1.36, 1.71, 2.04 min), respectively, were tentatively identified as 1-pyrroline-5-carboxylic acid and its isomers. They exhibited ESI-MS^1^ at *m*/*z* 114 [M+H]^+^. MS^2^ showed daughter ions at *m*/*z* 96 [M+H-H_2_O], 68 [M-COOH] corresponding to the loss of a water molecule and carboxyl groups, respectively. These data are in good agreement with the available literature [16].

Compound **14** (R_t_. 1.54 min) showed a protonated molecular ion peak [M+H]^+^ at *m*/*z* 122 and a daughter ion at *m*/*z* 105 [M+H-NH_3_], corresponding to the loss of an amino group. By comparison with the literature, peak **14** was tentatively identified as L-Cysteine [16].

Compound **16** (R_t_. 1.79 min) showed a molecular ion peak at *m*/*z* 131 [M-H]^−^, and MS^2^ produced a highly abundant fragment ion at *m*/*z* 85 [M-H-COOH] corresponding to the loss of the carboxyl group. Another fragment at *m*/*z* 69 [85-NH_2_] corresponded to the loss of an amino group. This fragmentation pattern, when compared with the available literature, confirmed that compound **16** is asparagine [23].

Compound **21** (R_t_. 3.76 min) showed ESI/MS at *m*/*z* 202 [M+H]^+^, and a MS/MS fragment at *m*/*z* 128 revealed the loss of [NH_2_-CH-CO-OH], leaving [CH_2_S_2_O_3_H]. In addition, the fragment ion at *m*/*z* 156 indicated the loss of a carboxylic group, and another MS^2^ fragment ion at *m*/*z* 185 showed the loss of an NH_3_ moiety. These fragmentation patterns are in good agreement with those of *S*-sulfocysteine [16].

Compounds **22**, **23**, and **24** (R_t_. 4.03, 4.08, 4.10 min) were analyzed using ESI-MS/MS, which showed the protonated molecular ion peak at *m*/*z* 144 [M+2H]^+^ and a product ion generated by the loss of a water molecule at *m*/*z* 126. Other MS/MS fragments at *m*/*z* 114 indicated the subsequent loss of CH_2_O. This fragmentation pattern is in good agreement with that of kojic acid [24]. Compounds **22**, **23**, and **24** were identified as kojic acid along with its two stereoisomers.

Compound **25** (R_t_. 4.27 min) was identified as *N*-acetyl L-cysteine from ESI/MS spectral data. It showed a molecular ion peak [M+H]^+^ at *m*/*z* 164. MS^2^ showed a fragment ion at *m*/*z* 121 corresponding to cysteine after the loss of the acetyl moiety. Additionally, daughter ions at *m*/*z* 102 [121-H_2_O-H] and 75 [121-COOH-H] were also observed. These fragmentation patterns are in good agreement with the literature of *N*-acetyl L-cysteine [16].

Compound **26** (R_t_. 5.45 min) generated the ESI-MS at *m*/*z* 130 [M+H]^+^, and MS^2^ fragmentation showed the base peak at *m*/*z* 84 [M+H-COOH], which was produced by the loss of the carboxyl group. The daughter ion at *m*/*z* 56 showed subsequent loss of CH_2_N moiety leaving C_4_H_8_. By comparison with the literature, compound **26** was tentatively identified as pipecolic acid [16].

Compound **28** (R_t_. 15.18 min) exhibited ESI-MS/MS at *m*/*z* 381 [M+H-H_2_O]^+^, corresponding to the loss of one water molecule. MS^2^ gave a daughter ion at *m*/*z* 274 [M+H- C_9_H_17_] corresponding to the loss of the side chain. These data, along with the aid of the relevant literature source [25], tentatively identified peak **28** as brassicasterol. Research implies that brassicasterol holds potential as a prospective compound for the development of drugs targeting HSV-1, tuberculosis, and ACE inhibition [26].

Compound **29** (R_t_. 29.69 min.) ESI/MS (positive mode) showed a pseudo-molecular ion at *m*/*z* 757.8 corresponding to the molecular formula C_43_H_83_NO_9_. The presence of a glucose moiety in this compound was evidenced by a fragment ion at *m*/*z* 595. Other fragment ions at *m*/*z* 85 (C_6_H_13_), 209 (C_15_H_29_), 239(C_16_H_31_O), and 281 (C_18_H_35_O) correspond to the sphingosine moiety [13]. The fragment *m*/*z* 459 indicates the loss of C_19_H_35_O_2_ for monohydroxylated saturated fatty acid. Another fragment at *m*/*z* 211 (C_15_H_31_) and 239 (C_17_H_35_) for a saturated fatty acid were observed. Another fragment showing the loss of the glucose moiety, hydroxyl group, and C_8_H_17_ was obtained at *m*/*z* 465. Fragment ions at *m*/*z* 228 (C_15_H_31_ + OH) were also detected. Based on this fragmentation pattern and comparison with the literature, peak 29 was concluded to be a cerebroside named hymedsmoside [13]. This compound was isolated in this work.

**Table 1:** Metabolites tentatively identified in *Hymedesmia* sp. ethyl acetate fraction using UPLC-ESI-MS/MS analysis in positive ionization mode.

No.	Compound Name	R_t_ (Min.)	Parent Ion (*m*/*z*)	MS^2^ Fragments (*m*/*z*)	Area% Total	Reference
1	* Uracil	0.21	114 [M+2H]^+^	70	7.12	[16]
2	Aminobutyric acid	0.74	104 [M+H]^+^	59, 44, 43	7.12	[16]
3	Threonine	0.68	120 [M+H]^+^	102 [M^+^-H_2_O], 74, 70	1.92	[17,18]
4	Threonine isomer	0.78	120 [M+H]^+^	102 [M^+^-H_2_O], 84, 74, 70	0.29	[17,18]
5	Kynurenine	0.78	209 [M+H]^+^	164, 136, 128, 118, 136, 94	0.90	[16,19]
6	Threonine isomer	1.88	120 [M+H]^+^	102 [M^+^-H_2_O], 74, 56	0.56	[17,18]
7	Uracil	0.27	113 MS^1^		0.95	[16]
8	* Adenine	0.80	137 [M+2H]^+^	119 [M^+^+H-NH_3_]^+^, 109, 93, 66	7.62	[16,20]
9	Adenine isomer	0.94	137 [M^+^+2H]^+^	119 [M^+^+H-NH_3_]^+^, 109, 93, 66	0.98	[20]
10	* Thymidine	1.06	241 [M-H]^−^ MS^1^	125 [thymine H]	6.95	[21]
11	* Thymine	1.18	127 [M+H]^+^	110, 84, 56, 44	4.91	[16]
12	L-phenylalanine	1.21	166 [M+H]^+^	120	0.75	[16]
13	1-Pyroline-5-carboxylic acid	1.36	114 [M+H]^+^	96 [M+H-H_2_O], 67 [M+H-COOH]	1.19	[16]
14	L-cysteine	1.54	122 [M+H]^+^	105 [M+H-NH_3_]	29.67	[16]
15	1-pyroline-5-carboxylic acid isomer	1.71	114 [M+H]^+^	96 [M+H-H_2_O], 67 [M+H-COOH]	0.35	[16]
16	Asparagine	1.79	131 [M-H]^−^	85 [M-H-COOH], 69 [85-NH_2_]	0.67	[23]
17	1-pyroline-5-carboxylic acid isomer	2.04	114 [M+H]^+^	96 [M+H-H_2_O], 68 [M-COOH]	0.25	[16]
18	Thymine isomer	2.06	127 [M+H]^+^	110, 84, 56, 44	0.38	[16]
19	Thymine isomer	2.10	127 [M+H]^+^	110, 84, 56, 44	0.54	[16]
20	Thymidine isomer	3.48	243 [M+H]^+^ 241 [M-H]^−^	125	0.65	[16]
21	*S*-sulfo-L-cysteine	3.76	202 [M+H]^+^	185, 156, 128, 73, 46	0.30	[16]
22	Kojic acid	4.03	144 [M+2H]^+^	126 [M^+^+2H-H_2_O], 115 [M^+^+2H-CHO], 90.8, 65.8	1.95	[24]
23	Kojic acid isomer	4.08	144 [M+2H]^+^	127 [M^+^+2H-H_2_O], 115 [M^+^+2H-CHO], 90.8, 65.8	0.63	[24]
24	Kojic acid isomer	4.10	144 [M+2H]^+^	127 [M^+^+2H-H_2_O], 115 [M^+^+2H-CHO], 90.8, 65.8	0.53	[24]
25	*N*-acetyl-L-cysteine	4.27	164 [M+H]^+^	121, 102, 75	0.24	[16]
26	Pipecolic acid	5.45	130 [M+H]^+^	84, 56	0.37	[16]
27	* Uridine	8.71	245 [M+H]^+^	132, 112, 55, 43	4.53	[27]
28	Brassicasterol	15.18	381[M+H-H_2_O]^+^	274, 255, 105, 95, 55	2.18	[25]
29	* Hymedesmoside	29.96	757.8 [M+H]^+^	595, 459, 281, 239, 209, 85	2.08	[13]

* Compounds isolated from ethyl acetate fraction.

### 2.3. Biological Activities of Hymedesmia sp. Ethyl Acetate (HE) Fraction

#### 2.3.1. Antimicrobial Activity and Minimum Inhibitory Concentration (MIC)

Bacterial resistance is an evolutionary process, even though antibiotics have proven to be highly effective in the treatment of bacterial infections. Bacteria can mutate and develop resistance mechanisms in response to the antibiotic’s selective pressure. This global issue is of significant concern. In this context, the discovery of new antimicrobials is not just a possibility but a necessity. Several antimicrobial compounds are plant-derived, emphasizing the importance of investigating natural resources for prospective approaches to control bacterial resistance and infectious diseases [28,29]. The marine ecosystem is a remarkable source of various natural products with a wide range of bioactivities. Among these, antimicrobial compounds exhibit significant activity against many drug-resistant bacteria and fungi, making marine natural products a highly promising source for the discovery of new antimicrobial drugs [30].

In the current study, the antimicrobial activity of the *Hymedesmia* sp. ethyl acetate fraction (HE) was investigated for the first time against *Staphylococcus aureus* ATCC 5368 (Gram-positive bacteria), *Escherichia coli* ATCC 10536, *Pseudomonas aeruginosa* ATCC 27853 (Gram-negative bacteria), and *Candida albicans* ATCC 10231 (fungus), in addition to determining the minimum inhibitory concentration (MIC).

The antimicrobial activity of the tested fraction was classified based on the MIC values as follows: 50–500 µg/mL = strong activity; 600–1500 µg/mL = moderate activity; and >1500 µg/mL = weak activity or inactive [31].

**Figure 5:**
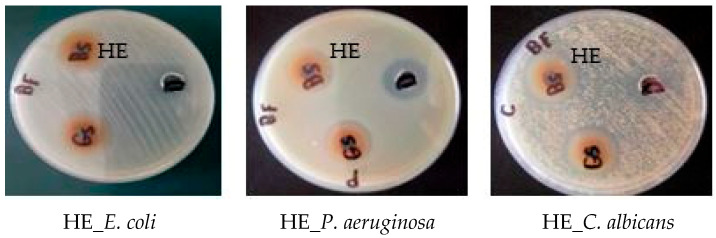
Antimicrobial activity of *Hymedesmia* sp. ethyl acetate (HE) fractions by agar dilution method against *S. aureus*, *E. coli*, *P. aeruginosa* and *C. albicans*.

According to the previous classification, *Hymedesmia* sp. ethyl acetate (HE) fraction exhibited strong antibacterial activity against *S. aureus* only with a MIC value of 62.5 ± 0.88 µg/mL, compared to ciprofloxacin (positive control) with a MIC value of 1.56 ± 1.2 for Gram-positive bacteria. It also showed strong antibacterial activity against *E. coli* and *P. aeruginosa* with MIC values of 125 ± 0.98 and 31.25 ± 0.32 µg/mL, respectively, compared to the positive control with a MIC value of 3.125 ± 0.89 for Gram-negative bacteria. However, it exhibited weak or inactive activity against *C. albicans* (Figure 5, Table 2).

The significant antimicrobial activity may be attributed to the presence of pipecolic acid [33], kojic acid [34], and cerebroside (hymedesmoside) [35], which have potent antimicrobial properties.

#### 2.3.2. Antioxidant Activity

Recently, several research studies have shown that free radicals are the main cause of oxidative damage to biomolecules and nucleic acids. To maintain health, the balance between free radicals and antioxidants is essential. Therefore, controlling oxidative stress may be a must in the prophylaxis and treatment of many serious diseases [36].

In light of the growing interest of the food and pharmaceutical industries to develop natural, biologically active antioxidants for the management of various diseases such as cancer and age-related disorders, antioxidant activity has become a hot topic of intense research [37,38]. The marine environment has been recognized as a rich supply of various bioactive compounds, such as antioxidants and antimicrobials [39].

The present study used the DPPH method to examine the antioxidant activity of the ethyl acetate (HE) fractions of *Hymedesmia* sp. marine sponge. Ascorbic acid was used as a positive control.

**Figure 6:**
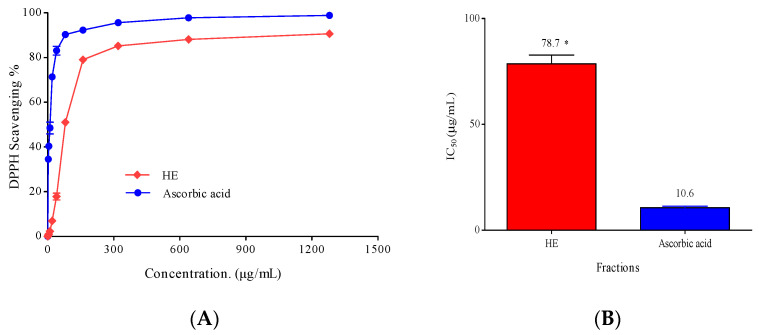
(**A**) 2,2-diphenyl-picrylhydrazyl (DPPH) radical scavenging activity of different concentrations (5–1280 µg/mL) of *Hymedesmia* sp. ethyl acetate (HE) fractions (**B**) IC_50_ of antioxidant activity of *Hymedesmia* sp. ethyl acetate (HE) fractions and ascorbic acid (positive control). DPPH in methanol (without the tested sample) was used as a negative control. Data were analyzed using one-way ANOVA, and statistical significance was calculated with Dunnett’s multiple comparisons test. Significance level compared to the control is indicated by asterisks (*, *p* < 0.05). The data display the mean ± SD of three biological replicas.

The HE fraction showed a concentration-dependent antioxidant activity, as displayed in Figure 6A, by an increase in its DPPH radical scavenging percentage. The concentration required to scavenge DPPH by 50% (IC_50_ value) is shown in Figure 6B. Remarkably, the higher the scavenging activity, the smaller the IC_50_. Generally, the tested fraction is considered a weak antioxidant when IC_50_ values are between 151 and 200, moderate between 100 and 150, strong between 50 and 100, and very strong when IC_50_ values are less than 50, as reported by [40].

Based on the previous assortment, the ethyl acetate fraction (HE) displayed a strong DPPH scavenging activity with an IC_50_ of 78.70 ± 4.06 µg/mL compared to ascorbic acid as a positive control with an IC_50_ of 10.6 ± 0.8 µg/mL. Possibly, the ethyl acetate fraction’s antioxidant activity is due to the presence of sulfur containing amino acids such as cysteine, whose thiol group is involved in oxidation–reduction reactions. It possesses antioxidant properties like the ability to scavenge free radicals and chelate metals [41]. Brassicasterol and kojic acid also show potent antioxidant activities [6,42]. The antioxidant activity of marine sterols has been demonstrated by their ability to normalize several oxidative indicators and to initiate the expression of enzymatic and non-enzymatic antioxidants. Furthermore, they resemble biological sterols, particularly cholesterol, in both structure and function. Recent developments in technology, such as nanoparticles and microencapsulation, suggest that marine sterols could be useful lead compounds to produce novel therapeutic agents [42,43].

#### 2.3.3. Cytotoxic Activity

Cancer is one of the most destructive and detrimental diseases worldwide and poses a significant challenge in terms of treatment. It is the leading cause of death, with over 10 million deaths in 2020. By 2040, an expected 28.4 million cases of cancer are expected worldwide. Cancer treatment has many limitations in terms of efficacy, associated side effects, and high treatment costs. Therefore, there is an urgent need to develop new cancer therapies to address these challenges. Marine natural drugs are valuable sources of various secondary metabolites with impressive bioactivities including antimicrobial, antioxidant, anti-inflammatory, antihypertensive, antidiabetic, antiparasitic in addition to anticancer activity [44,45]. Interest in exploring various bioactivities of sponges has increased recently due to the identification of toxic compounds from various sponge species [46].

The cytotoxic activity of the *Hymedesmia* sp. ethyl acetate (HE) fraction was determined by 3-[4,5-dimethylthiazol-2-yl]-2,5 diphenyl tetrazolium bromide (MTT) assay against larynx carcinoma (HEP-2) and colon carcinoma (HCT-116) cell lines (obtained from VACSERA Tissue Culture Unit Giza, Egypt) over a concentration range of 0–500 µg/mL. Based on the IC_50_ values, the potency of the cytotoxic substances was classified as follows: IC_50_ ≤ 20 µg/mL is highly active, IC_50_ 21–200 µg/mL is moderately active, IC_50_ 201–500 µg/mL is weakly active, and IC_50_ > 501 µg/mL is inactive, which is in good agreement with the American National Cancer Institute protocol [47].

**Figure 7:**
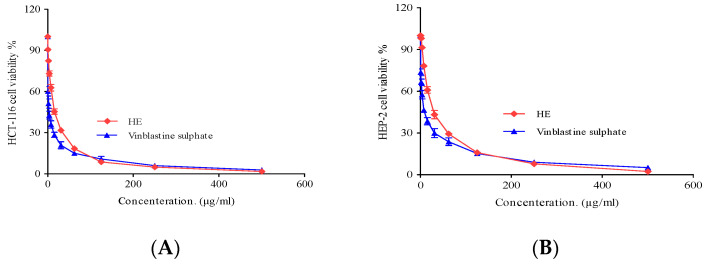
(**A**) Cytotoxic activity of *Hymedesmia* sp. ethyl acetate (HE) fraction against HCT-116 cell line at different concentrations. DMSO and vinblastine sulphate were used as negative and positive controls, respectively. (**B**) Cytotoxic activity of *Hymedesmia* sp. ethyl acetate (HE) fractions against HEP-2 cell line at different concentrations. Data were analyzed using one-way ANOVA, and statistical significance was calculated with Dunnett’s multiple comparisons test. A *p*-value < 0.05 was considered statistically significant. The data display the mean ±SD of three biological replicates.

The tested HE fraction, as shown in Figure 7, exhibited remarkable cytotoxic activity against the investigated cell lines with IC_50_ ranging from 13.5 ± 0.8 to 182.1 ± 6.75 µg/mL for HCT-116 and 25.3 ± 2.4 to 212.2 ± 8.13 µg/mL for HEP-2. Notably, the *Hymedesmia* sp. ethyl acetate fraction (HE) showed significant cytotoxic activity with IC_50_ values of 13.5 ± 0.8 µg/mL for HCT-116 and 25.3 ± 2.4 µg/mL for HEP-2 cell lines, respectively, compared to vinblastine (positive control) with IC_50_ values of 2.34 ± 0.28 µg/mL for HCT-116 and 6.61 ± 0.59 µg/mL for HEP-2. The tested ethyl acetate fraction, as shown in Table 3, exhibited moderate cytotoxic activity [47].

The ethyl acetate fraction (HE) analysis by UPLC-MS/MS revealed the presence of several compounds with reported anticancer activity, particularly cerebroside (hymadesmoside) and ceramides (bioactive lipids) found in various marine organisms. These compounds are involved in various physiological functions, including cell growth inhibition, apoptosis, cell growth arrest, and cell aging. Additionally, they are known to be precursors of complex sphingolipids. Ceramides displaying cytotoxic activity have been previously isolated from marine sponges [48]. In addition, purine nucleobases such as adenine, pyrimidine nucleobases such as uracil and thymine, and nucleoside uridine have received much attention for their anticancer properties [49]. Moreover, kojic acid has shown anticancer activity [50]. Several studies have also reported the antimigratory and antiproliferative properties of kynurenine [51]. Additionally, pipecolic acid enhances resistance to bacterial infection.

## 3. Materials and Methods

### 3.1. General Materials and Methods

Evaporation of the solvents was achieved using a Buchi rotary evaporator. A UV lamp was used for thin-layer chromatography (TLC) visualization: UVP, GL-58 (λ_max_ 254 and 366 nm). A circulating hot-air oven, WT-Binder 7200 (Germany), was used in this study. Infrared (IR) spectral analysis was recorded in potassium bromide disks on a Pye Unicam SP 3000 and an IR spectrophotometer (FT/IR-460 plus; Jasco).

UPLC-ESI-MS/MS in positive ionization mode was performed on a XEVO-TQD triple-quadrupole instrument (Waters Corporation, Milford, MA, USA) mass spectrometer: Column, ACQUITY UPLC BEH C_18_ 1.7 mm, 2.1 × 50 mm; column flow rate, 0.2 mL/min; solvent system consisted of (A) water containing 0.1% formic acid, (B) methanol containing 0.1% formic acid (Ain Shams University, Cairo, Egypt). Chromatographic separation was performed using a binary LC solvent system controlled by MassLynx (version 4.1) for the analysis of MS and MS2 data. The gradient, as described by [6,52], is as follows: 0–2 min 10% B isocratic; 2–5 min, linear gradient B 10 to 30%; 5–15 min, linear gradient from 30% to 70% B; 15–22 min, linear gradient from 70 to 90% B; 22–25 min, 90% B isocratic. Washing and reconditioning of the column are included. Source temperature 150 °C (temperature); 30 eV (cone voltage); 3 kV (capillary voltage); 440 °C (desolvation temperature); 50 L/h (cone gas flow); and 900 L/h (desolvation gas flow) were the adjusted ESI parameters in the positive ionization mode. The MS^2^ collision energy settings were maintained at 30 eV.

Nuclear magnetic resonance (NMR) experiments 1D and 2D were carried out using a Bruker AMX 400 MHz for ^1^H NMR and with standard pulse sequences operating at 100 MHz for ^13^C NMR. ^1^H-^13^C one-bond connectivity was detected with the HSQC gradient pulse factor selection. Two- and three-bond connectivity was identified by the HMBC experiment. Coupling constants (*J*) are reported in Hz, and chemical shifts are reported in *δ* (ppm), unless otherwise mentioned. The internal standard used was Tetramethyl silane. Spectroscopic grade DMSO_*d_6_* (solvent at room temperature) was used for spectral analysis. Equipment used included an autoclave (Medexport BK-75), pH meter (Hanna Instruments 85/9), and a laminar air flow cabinet (Dalton).

### 3.2. Collection of Marine Sponge Samples

**Figure 8:**
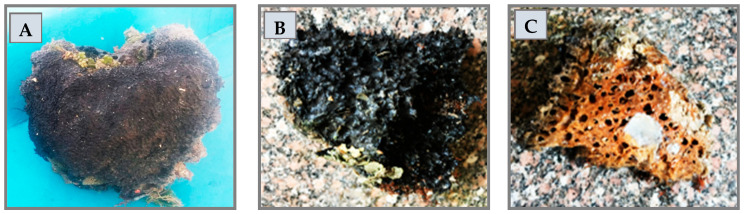
Pictures of *Hymedesmia* sp. sponge. Freshly collected specimen (**A**) and frozen specimen outer surface (**B**) and inner surface (**C**).

Marine sponge, namely, *Hymedesmia* sp. (Class *Demospongiae*), was collected from the Red Sea, near Sharm El Sheikh, Egypt [coordinates 27°45′57.8″ N 34°22′10.8″ E], at a depth of 8–10 m, during Nov–Dec/2018 using scuba diving (see Figure 8). The collected material was immediately frozen and kept at −20 °C until further investigation. Professor Saad Zakaria from the Marine Science Department, Faculty of Science, Suez Canal University, Egypt, identified the sponge biomass. A voucher specimen has been deposited in the natural product and pharmacognosy department, Faculty of Pharmacy, Zagazig University, under registration number EA-2018-101.

### 3.3. Extraction and Fractionation of Hymedesmia sp. Marine Sponge

It was carried out according to [41]. In brief, the fresh sponge material *Hymedesmia* sp. (5.25 kg wet weight) was frozen immediately after collection. The frozen sponge sample was minced into tiny pieces and shade dried for 48 h. The sample was subsequently extracted with absolute ethanol (3 × 6 L) at ambient temperature to exhaustion, filtered, and vacuum-evaporated to yield a viscous extract (270 g). The concentrated extract was then dispersed in water/methanol (9:1) and successively partitioned with *n*-hexane, dichloromethane, and ethyl acetate to obtain *n*-hexane (17 g), dichloromethane (6.18 g), and ethyl acetate (4 g) fractions as shown in Scheme S1.

### 3.4. Isolation of Compounds from the Ethyl Acetate Soluble Fraction of Hymedesmia sp.

About 3.5 g of the ethyl acetate-soluble fraction of the *Hymedesmia* sp. marine sponge was dissolved in the least amount of methanol and adsorbed on 4 g of silica gel. The solvent was evaporated to dryness at room temperature. The dried mixed initial zone was then placed on the top of a silica gel column (88 g, 110 × 3 cm). The eluate was collected in 93 fractions each of 250 mL, concentrated under vacuum, and monitored by TLC using a solvent system of ethyl acetate/methanol/water (6:1:0.8). TLC plates were visualized using Dragendorff’s reagent and/or vanillin/sulphuric acid.

#### 3.4.1. Isolation and Identification of Compound **1** (Thymine)

TLC screening of fractions eluted with 70% ethyl acetate *n*-hexane using the solvent system (dichloromethane:methanol 9.5:0.5), with Dragendorff’s and vanillin/sulphuric acid as visualizing reagents, revealed the presence of a major faint orange spot with an Rf value of 0.75. The fractions were pooled, evaporated, and crystallized with methanol to afford 8 mg of crystalline white powder designated as compound **1**. It is freely soluble in methanol and insoluble in n-hexane and dichloromethane.

The ^1^H-NMR and ^13^C-NMR (400 MHz and 100 MHz, DMSO) spectra for compound **1** (thymine) are summarized in Table S1 and Figures S3 and S4. ESI-MS/MS (Figure S1) showed a pseudo-molecular ion peak [M+H]^+^ at *m*/*z* 127, which is compatible with the molecular formula C_5_H_6_N_2_O_2_. The IR spectrum (Figure S2) revealed the presence of a sharp NH absorption peak at λ_max_ 3300 cm^−1^ and a peak at λ_max_ 2928 cm^−1^ corresponding to (C-H stretching). An intense sharp peak at λ_max_ 1652 cm^−1^ (C=O) indicates the presence of an amide group. Additionally, a peak at λ_max_ 1380 cm^−1^ corresponds to (-CH3) bending.

#### 3.4.2. Isolation and Identification of Compound **2** (Uracil)

TLC investigation of fractions eluted with 85% ethyl acetate *n*-hexane using the solvent system (dichloromethane/methanol, 9.5:0.5) and vanillin/sulphuric acid as a visualizing reagent revealed the presence of one major violet spot with an R_f_ value of 0.63. The fractions were collected, evaporated, and crystallized with methanol to give 10 mg of crystalline white powder named compound **2**. It is freely soluble in methanol and insoluble in *n*-hexane and dichloromethane.

The ^1^H-NMR and ^13^C-NMR (400 MHz and 100 MHz, DMSO) for compound **2** (uracil) are summarized in Table S2 and shown in Figures S7 and S8. HSQC and HMBC are shown in Figures S9 and S10, respectively. ESI-MS/MS (Figure S5) showed a pseudo-molecular ion peak [M+H]^+^ at *m*/*z* 113 [M+H]^+^, which was compatible with the molecular formula C_4_H_4_N_2_O_2_. The IR spectrum in Figure S6 revealed the presence of a sharp NH absorption peak at λ_max_ 3382 cm^−1^ and intense sharp peaks at λ_max_ 1714 and 1634 cm^−1^ of the carbonyl and amide group, respectively.

#### 3.4.3. Isolation and Identification of Compounds **3** and **4** (Thymidine and Uridine)

Screening of the fraction eluted with 100% ethyl acetate using the solvent system (ethyl acetate/methanol/water, 6:1:0.8) with Dragendorff’s reagent as a visualizing reagent revealed the presence of one major faint orange spot with an Rf value of 0.61 and one minor faint orange spot with an Rf value of 0.63. The fractions were collected, concentrated under reduced pressure, and crystallized using hot methanol to yield 16 mg of crystalline white clusters designated as compounds (**3** and **4**). The compounds are freely soluble in methanol and insoluble in n-hexane and dichloromethane.

The ^1^H-NMR and ^13^C-NMR (400 MHz and 100 MHz, DMSO) spectra for compounds **3** and **4** (thymidine and uridine) are summarized in Table S3 and shown in Figures S16 and S17. HSQC and HMBC spectra are shown in Figures S18 and S19, respectively. ESI-MS in the positive mode (Figure S11) showed prominent peaks at *m*/*z* 243 and 245, which can be assigned to the molecular formulas C_10_H_14_N_2_O_5_ and C_9_H_12_N_2_O_6_, respectively, and proposed that this is a mixture of two pyrimidine nucleosides.

The positive ESI-MS spectrum of compound **3** (Figure S12) showed a protonated molecular ion peak at *m*/*z* 243 [M+H]^+^ and daughter ions at *m*/*z* 200 [M+H-CONH] indicating the loss of the amide moiety. Additionally, the fragment ion at *m*/*z* 169 suggests the subsequent loss of the CH_2_-OH moiety.

The ESI-MS/MS spectrum of compound **3** in negative mode (Figure S13) exhibited a molecular ion peak at *m*/*z* 287 [M+H+HCOOH] and another molecular ion peak at *m*/*z* 241[M-H]^−^. Furthermore, the fragment ion at *m*/*z* 125 corresponded to [M^+^-H-deoxy ribose] of thymine after the loss of the deoxyribose moiety. In addition, the ESI MS/MS spectrum of compound **4** in positive mode (Figure S11) showed a protonated molecular ion peak at *m*/*z* 245 [M+H]^+^ and daughter ions at *m*/*z* 132 and 112 corresponding to ribose sugar and uracil moieties, respectively. Furthermore, other fragment ions at 55 (C_3_H_2_O) and 42 (CHNO) were also observed.

The IR spectral spectrum (Figure S15) of compounds **3** and **4** indicated the presence of an intense sharp peak at λ_max_ 3301 cm^−1^ corresponding to the (OH) group. The absorption bands between λ_max_ 2961 and 2870 cm^−1^ correspond to the stretching vibrations of C-H aliphatic. The sharp peak appearing at λ_max_ 1653 cm^−1^ belongs to the carbonyl C=O of the amide group.

#### 3.4.4. Isolation and Identification of Compound **5** (Adenine)

Fractions eluted with 1% methanol/ethyl acetate were screened using TLC with a solvent system of ethyl acetate/methanol/water (6:1:0.8) and Dragendorff’s reagent as a visualizing reagent. This revealed the presence of one major faint orange spot with an Rf value of 0.41. The fractions were then combined, concentrated, and crystallized with methanol resulting in 9 mg of cluster crystals designated as compound **5**. Compound **5** is freely soluble in methanol and insoluble in *n*-hexane and dichloromethane.

The ^1^H-NMR (400 MHz, DMSO) for compound **5** (adenine) is summarized in Table S4 and Figure S22. ESI-MS/MS (Figure S20) showed a pseudo-molecular ion peak at *m*/*z* 137 [M+2H]^+^, which is compatible with the molecular formula C_5_H_5_N_5_. The presence of adenine was supported by the remaining ESI/MS/MS fragments. The IR spectrum of compound **5** (Figure S21) showed absorption bands at λ_max_ 3300 and 3350 cm^−1^ for primary and secondary amine groups. The peak at λ_max_ 1663 cm^−1^ indicated the presence of a C=C group.

#### 3.4.5. Isolation and Identification of Compound **6** (Hymedesmoside)

TLC screening of fractions eluted with 3% methanol/ethyl acetate using the solvent system (ethyl acetate/methanol/water (6:1:0.8) and vanillin/sulphuric acid as a visualizing reagent revealed the presence of one major blue spot with an Rf value of 0.32. The fractions were pooled, evaporated, and crystallized with methanol to afford 17 mg of off-white powder designated as compounds **6**. It is freely soluble in a mixture of dichloromethane and methanol.

The ^1^H-NMR and ^13^C-NMR (400 MHz and 100 MHz, DMSO) for compound **6** (Hymedesmoside) are shown in (Figures S26–S28). ESI/MS/MS (positive mode) (Figure S24) showed a pseudo-molecular ion at *m*/*z* 757.8 corresponding to the molecular formula C_43_H_83_NO_9_. IR spectrum of compound **6** (Figure S23) revealed the presence of OH and NH absorption peaks at λ_max_ 3297 and 3330 cm^−1^, respectively. Peaks at λ_max_ 2917 and 2850 cm^−1^ indicated C-H stretching. In addition, there was an absorption peak at 1651 cm^−1^ for the C=C group.

### 3.5. Biological Activities

The antioxidant, cytotoxic, and antimicrobial activities of the *Hymedesmia* sp. ethyl acetate fraction were investigated at the Regional Center for Mycology and Biotechnology (RCMB) at Al-Azhar University, Egypt.

#### 3.5.1. Antioxidant Activity

The antioxidant activity of the *Hymedesmia* sp. HE fraction was assessed by the DPPH free radical scavenging assay as described by [6,53,54]. In summary, 2,2-diphenyl-1-picrylhydrazyl radical (DPPH) was freshly prepared in methanol (0.004% *w*/*v*) and stored in the dark at 10 °C.

Different concentrations (2.5, 5, 10, 20, 40, 80, 160, 320, 640, and 1280 µg/mL) of the tested fraction in methanol were also prepared. A forty µL aliquot of the methanol solution was added to 3 mL of DPPH solution and allowed to stand for 10 min at room temperature in the dark. Absorbance measurements were recorded immediately with a UV–visible spectrophotometer (Milton Roy, Spectronic 1201, Houston, TX, USA). The decrease in absorbance at λ_max_ 515 nm was measured continuously, with data being recorded at 1 min intervals until the absorbance stabilized (16 min). The absorbance of the DPPH radical without an antioxidant (negative control) and the reference compound ascorbic acid (positive control) were also measured. All the calculations were performed in triplicates and averaged. Using the following formula, the percentage inhibition (PI) of the DPPH radical was calculated,

PI = [(*A*C − *A*T)/*A*C] × 100

where *A*C = Absorbance of the control at t = 0 min and *A*T = absorbance of the sample + DPPH at t = 16 min [55]. The antioxidant capacity (IC_50_), which causes a 50% decline in the absorbance of the DPPH solution from its starting absorbance, was measured by plotting the DPPH radical scavenging percentage against each sample concentration and ascorbic acid (µg/mL). The antioxidant activity increases with a decreasing IC_50_.

#### 3.5.2. Cytotoxic Activity

*Hymedesmia* sp. HE fraction was evaluated for its cytotoxic activity against HEB-2 and HCT-116 (human larynx carcinoma and colon carcinoma) cell lines using MTT (3-(4, 5-dimethylthiazole-2-yl)-2, 5-diphenyl-tetrazolium bromide) assay as reported by [6,56,57]. This is a colorimetric assay that depends on the ability of mitochondrial reductase from living cells to transform the yellow MTT (water-soluble dye) into insoluble purple formazan crystals. The formazan is then dissolved, and the optical density at 490 nm is used to determine the concentration. The number of viable cells is directly proportional to the amount of soluble purple formazan [58]. Vinblastine sulphate was employed as a positive control, while Dimethyl sulfoxide (DMSO) was used as a negative control. The VACSERA Tissue Culture Unit provided the examined cell lines. Dulbecco’s Modified Eagle’s Medium, or DMEM, was utilized to propagate the examined cells. The ethyl acetate fraction was prepared as stock solutions in 10% DMSO in ddH_2_O. Cytotoxicity was assessed using the MTT test as described by [6,57]. In summary, 96-well plates were seeded with 100 µL of cells per well at a density of 1 × 10^4^ cells/mL. The plates were then incubated for 24 h at 37 °C with 5% CO_2_. After 24 h, cells were treated in triplicate using different amounts of the tested fraction. The viability of cells was measured using a colorimetric technique. The supernatant was removed after another 24 h, and each well was then filled with 1% crystal violet solution for at least 30 min. After the stain was removed, the plates were thoroughly cleaned with tap water to eliminate any remaining residue. Each well was then filled with 30% glacial acetic acid and then stirred. The absorbance of the plates was measured at 490 nm on a Microplate reader (TECAN, Inc.., Morrisville, NC, USA). The number of viable cells was determined by measuring the optical density with the microplate reader (SunRise, TECAN, Inc., USA). The following equation was used to determine the viability %:

Cell viability % = [1 − (ODt/ODc)] × 100%

where ODt is the mean optical density of wells treated with the tested fraction and ODc is the mean optical density of untreated cells.

The survival curve of each tumor cell line following treatment with the ethyl acetate fraction is obtained by plotting the relationship between remaining cells and drug concentration. The concentration which produces adverse effects in 50% of intact cells (IC_50_) was determined from graphic plots of the dose–response curve for each concentration using GraphPad Prism software (San Diego, CA, USA).

#### 3.5.3. Antimicrobial Activity

The antibacterial activity of the *Hymedesmia* sp. ethyl acetate fraction was evaluated using the well diffusion method as described by [6,59] against *Escherichia coli* (*E. coli*, ATCC 10536) and *Pseudomonas aeruginosa (P. aeruginosa*, ATCC 27853) as Gram-negative bacteria and against *Staphylococcus aureus* (*S. aureus*, ATCC 5368) as Gram-positive bacteria. Ciprofloxacin (100 µg/mL) was used as the standard (positive control). The antibacterial activity was calculated by measuring the diameter of the inhibition zone in mm from three independent experiments, and the average was taken. The antifungal activity was also assessed against *Candida albicans* (*C. albicans,* ATCC 10231) as reported by [6]. Fluconazole (100 µg/mL) was used as the positive antifungal control. The tested fraction was dissolved in dimethyl sulfoxide (DMSO) at a concentration of 500 µg/mL. DMSO was used as a negative control. Potato Dextrose Agar (PDA) medium was used for *C. albicans*, whereas Muller Hinton Agar (MHA) medium was used for bacterial strains. Each sample’s stock solution (100 µL) was added to the wells, and the standards and DMSO cultures were incubated for 24–48 h for fungus and 14–18 h for bacteria at 37 °C. Al-Azhar University in Egypt’s Regional Center for Mycology and Biotechnology (RCMB) provided the tested bacterial strains and *Candida albicans* [60,61].

##### Minimum Inhibitory Concentration Determination (MIC) 

The MIC values of the *Hymedesmia* sp. ethyl acetate fraction were determined using the agar dilution method, as described in [5]. In summary, the (HE) fraction was serially diluted after being dissolved in DMSO at concentrations of no more than 5% and 2.5% for fungus and bacteria, respectively. For the investigated bacterial strains, a series of MHA plates and PDA plates were constructed, each containing varying dilutions of the tested fraction. After growing the investigated bacterial strains on MHA overnight, the purified colonies were suspended in 0.9% saline. After adjusting the turbidity of the bacterial inoculum to 0.5 McFarland standard (2.5 × 10^8^ cfu/mL), it was diluted to 1:10 using sterile saline. A final concentration of 10^4^ cfu per spot was achieved by inoculating pre-prepared MHA plates with 2 µL of the prepared inoculum applied to their surfaces [62].

*C. albicans* was plated on PDA. The purified colonies were suspended in saline. The turbidity was adjusted to 0.5 McFarland standard (5 × 10^6^ cfu/mL) and, then, diluted 1:10 with saline. PDA containing different concentrations of the HE fraction was prepared and inoculated by adding 2 µL of the prepared inoculum, resulting in a final inoculum concentration of 10^3^ per spot. Inoculated plates kept at 30 °C for 24–48 h were examined for the presence of microbial growth. MIC is the lowest concentration of the antimicrobial agent that inhibits the growth completely [63,64].

### 3.6. Statistical Analysis

The collected data were plotted using GraphPad Prism 5 software (GraphPad Software, San Diego, CA, USA). Data analysis and statistical significance calculations were conducted using one-way ANOVA followed by Dunnett’s multiple comparisons test. A *p*-value < 0.05 was considered statistically significant. The data represent the mean ± SD of three biological replicates.

## 4. Conclusions

The *Hymedesmia* sp. ethyl acetate (HE) fraction was analyzed for the first time using UPLC-ESI-MS/MS analysis, revealing the tentative identification of twenty-nine compounds. Isolation and structure determination of thymine, uracil, thymidine, uridine, adenine, and hymedesmoside (cerebroside) from the ethyl acetate fraction as major components were conducted. The cytotoxic, antioxidant, and antimicrobial activities were also assessed in vitro. Notably, the ethyl acetate fraction showed the most potent cytotoxic activity.

In conclusion, this is the first study to investigate the chemical composition and biological activities of the ethyl acetate fraction of *Hymedesmia* sp. sponge from the Red Sea, Egypt. Based on the previous results, this fraction could be a promising source of potent cytotoxic, antioxidant, and antimicrobial natural agents for resistant bacterial and fungal strains as well as different cancers. Further studies are planned to clarify the mechanism of action of this fraction as antimicrobial, anticancer, and antioxidant. Molecular docking of the isolated compounds will be of great value in understanding the mechanism of binding with the right targets and in designing new biologically active drugs.

## Figures and Tables

**Table 2:** Antimicrobial activity of *Hymedesmia* sp. ethyl acetate (HE) fraction by agar diffusion method.

Microorganism/ Fraction		Inhibition Zone (IZ mm) Diameter (mean ± SD)/Minimum Inhibitory Concentration (MIC µg/mL)							
		Gram Positive Bacteria		Gram Negative Bacteria				Fungus	
		*Staphylococcus aureus* ATCC 5368		*Escherichia coli* ATCC 10536		*Pseudomonas aeruginosa* ATCC 27853		*Candida albicans* ATCC 10231	
		IZ	MIC	IZ	MIC	IZ	MIC	IZ	MIC
*Hymdesmia* sp.	Ethyl acetate (HE)	22 ± 0.69	62.5 ± 0.88	22 ± 0.83	125 ± 0.98	21 ± 0.62	31.25 ± 0.32	25 ± 0.59	3000 ± 0.38
	Ciprofloxacin	-	1.56 ± 1.2	-	3.125 ± 0.89	-	3.125 ± 0.24	-	-
	Fluconazole	-	-	-	-	-	-	42 ± 0.58	50 ± 0.24
	DMSO (Solvent)	10		10		19		12	

MIC: 50–500 μg/mL (Strong activity), 600–1500 μg/mL (Moderate activity), >1500 μg/mL (Weak activity) [31,32].

**Table 3:** Half-maximum inhibitory concentration (IC_50_) of *Hymedesmia* sp. Ethyl acetate (HE) fractions in cell viability of HCT-116 and HEP-2 cells after treatment for 48 h, as measured by MTT assay. The data are presented as µg/mL.

Cell Line	IC_50_ (µg/mL)	
	Tested Ethyl Acetate Fraction (HE) of *Hymedesmia* sp.	
	HE	Vinblastine Sulphate
HCT-116 (Colon carcinoma)	13.5 ± 0.8	2.34 ± 0.28
HEP-2 (Human Larynx carcinoma)	25.3 ± 2.4	6.61 ± 0.59

These are the means of three determination of *Hymedesmia* sp. ethyl acetate (HE) fraction.

## Data Availability

Data is contained within the article.

## Supplementary Materials

The following supporting information can be downloaded at: https://www.mdpi.com/article/10.3390/ph17060724/s1, Scheme S1. Extraction and isolation of *Hymedesmia* sp. marine sponge; Table S1. ^1^H-NMR and ^13^C-NMR (400 MHz and 100 MHz, DMSO) of compounds **1** (Thymine), Table S2. ^1^H-NMR and ^13^C-NMR (400 MHz and 100 MHz, DMSO) spectral data for compound **2** (Uracil), Table S3. ^1^H-NMR and ^13^C-NMR (400 MHz and 100 MHz, DMSO) spectral data for compounds **3** and **4** (Thymidine and Uridine), Table S4. ^1^H-NMR (400 MHz, DMSO) spectral data for compound **5** (adenine); Figure S1. UPLC-ESI-MS/MS chromatogram of compounds **1** (Thymine), Figure S2. IR spectrum compound 1 (Thymine), Figure S3. ^1^H NMR spectrum compound 1 (Thymine), Figure S4. ^13^C NMR spectrum compound 1 (Thymine), Figure S5. UPLC-ESI-MS/MS chromatogram of compound **2** (Uracil), Figure S6. IR spectrum of compound **2** (Uracil), Figure S7. ^1^HNMR spectrum of compound **2** (Uracil), Figure S8. ^13^C NMR spectrum of compound **2** (Uracil), Figure S9. HSQC spectrum of compound **2** (Uracil), Figure S10. HMBC spectrum of compound **2** (Uracil), Figure S11. (A): UPLC-ESI-MS chromatogram of compounds **3** and **4** (Thymidine and Uridine) mixture in positive (+) ionization mode, (B): UPLC-ESI-MS/MS chromatogram of compounds **3** and **4** (Thymidine and Uridine) mixture in positive (+) ionization mode, Figure S12. UPLC-ESI-MS chromatogram of compound **3** thymidine (major) in positive (+) ionization mode, Figure S13. UPLC-ESI-MS chromatogram of compound **3** thymidine (major) in negative (−) ionization mode, Figure S14. UPLC-ESI-MS chromatogram of compound **4** uridine (minor) in negative (+) ionization mode, Figure S15. IR spectrum of compounds **3** and **4** (Thymidine and Uridine), Figure S16. ^1^HNMR spectrum of compounds **3** and **4** (Thymidine and Uridine), Figure S17. ^13^CNMR spectrum of compounds **3** and **4** (Thymidine and Uridine), Figure S18. HSQC spectrum of compound **3** and **4** (Thymidine and Uridine), Figure S19. HMBC spectrum of compounds **3** and **4** (Thymidine and Uridine), Figure S20. ESI-MS/MS/MS spectrum of compound **5** (adenine), Figure S21. IR spectrum of compound **5** (adenine), Figure S22. ^1^HNMR spectrum of compound **5** (adenine), Figure S23. IR spectrum of compound **6** (Hymedesmoside), Figure S24. Positive ESI-MS/MS spectrum of compound **6** (Hymedesmoside), Figure S25. ^1^H NMR spectrum (A), and (B and C) expansion spectrum of compound **6** (Hymedesmoside), Figure S26. ^13^C NMR spectrums of compound **6** (Hymedesmoside), Figure S27. ^1^HNMR and ^13^CNMR data of compound **6** (Hymedesmoside).
