# Supplementary material for: Chemical and Biological Characterization of the Ethyl Acetate Fraction from the Red Sea Marine Sponge Hymedesmia sp
Source: Pharmaceuticals (Basel). 2024 Jun 3;17(6):724. doi: 10.3390/ph17060724 (PMC11206279; doi:10.3390/ph17060724)
Supplement: Supplementary file 1 [file pharmaceuticals-17-00724-s001.zip › pharmaceuticals-3011865-supplementary.pdf]

Supplementary materials

Chemical and Biological Characterization of the Ethyl Acetate Fraction from the Red Sea Marine Sponge *Hymedesmia* sp.

Zeinab I. El Sayed [1], Wafaa H. B. Hassan [1], Mahmoud M. Abdel-Aal [1], Shaza M. Al-Massarani [2], Wael M. Abdel-Mageed [2,*], Omer A. Basudan [2], Mehtab Parveen[3], Eman Abdelsalam [1], and Sahar Abdelaziz [1,*]

[1]Department of Pharmacognosy, Faculty of Pharmacy, Zagazig University, 44519 Zagazig, Egypt; zinab_elsayed@su.edu.eg (Z.I.E), wafaahbh@zu.edu.eg (W.H.B.H); Mahmoud.ibrahim@su.edu.eg (M.M.A.-A.); eaamer@zu.edu.eg (E.A); sah_abdelaziz@zu.edu.eg (S.A.)

[2]Department of Pharmacognosy, College of Pharmacy, King Saud University, P.O. Box 2457, Riyadh 11451, Saudi Arabia; wabdelmageed@ksu.edu.sa (W.M.A.-M.)

[3]Department of Chemistry, Faculty of Science, Aligarh Muslim University, Aligarh 202002 (U.P.), India.

* Correspondence: wabdelmageed@ksu.edu.sa (W.M.A.); sah_abdelaziz@zu.edu.eg (S.A.)

List of contents

Scheme (1): Extraction and isolation of *Hymedesmia* sp. marine sponge
Table S1. ^{1}H-NMR & ^{13}C-NMR (400 MHz and 100 MHz, DMSO) of compounds 1 (thymine).
Table S2. ^{1}H-NMR and ^{13}C-NMR (400 MHz and 100 MHz, DMSO) spectral data for compound 2 (uracil).
Table S3. ^{1}H-NMR and ^{13}C-NMR (400 MHz and 100 MHz, DMSO) spectral data for compounds 3 & 4 (thymidine & uridine).
Table S4.^{1}H-NMR (400 MHz, DMSO) spectral data for compound 5 (adenine)

Figure S1. UPLC-ESI-MS/MS chromatogram of compounds 1 (thymine).
Figure S2. IR spectrum compounds 1 (thymine).
Figure S3. ^{1}H NMR spectrum compounds 1 (thymine).
Figure S4. ^{13}C NMR spectrum compounds 1 (thymine).
Figure S5. UPLC-ESI-MS/MS chromatogram of compound 2 (uracil).
Figure S6. IR spectrum of compound 2 (uracil).
Figure S7. ^{1}HNMR spectrum of compound 2 (uracil).
Figure S8. ^{13}C NMR spectrum of compound 2 (uracil)
Figure S9. HSQC spectrum of compound 2 (uracil)
Figure S10. HMBC spectrum of compound 2 (uracil)
Figure S11. (A): UPLC-ESI-MS chromatogram of compounds 3 & 4 (thymidine & uridine) mixture. in positive (+) ionization mode. (B): UPLC-ESI-MS/MS chromatogram of compounds 3 & 4 (thymidine & uridine) mixture in positive (+) ionization mode
Figure S12. UPLC-ESI-MS chromatogram of compound 3 thymidine (major) in positive (+) ionization mode.
Figure S13. UPLC-ESI-MS chromatogram of compound 3 thymidine (major) in negative (-) ionization mode.
Figure S14. UPLC-ESI-MS chromatogram of compound 4 uridine(minor) in negative (+) ionization mode.
Figure S15. IR spectrum of compounds 3 &4 (thymidine & uridine)
Figure S16. ^{1}HNMR spectrum of compounds 3 & 4 (thymidine & uridine)
Figure S17.^{13}CNMR spectrum of compounds 3 & 4 (thymidine & uridine)
Figure S18. HSQC spectrum of compound 3 & 4 (thymidine & uridine)
Figure S19. HMBC spectrum of compounds 3 & 4 (thymidine & uridine)
Figure S20. ESI-MS/MS/MS spectrum of compound 5 (adenine)
Figure S21. IR spectrum of compound 5 (adenine)
Figure S22. ^{1}HNMR spectrum of compound 5 (adenine)
Figure S23. IR spectrum of compound 6 (Hymedesmoside)
Figure S24. Positive ESI-MS/MS spectrum of compound 6 (hymedesmoside)
Figure S25. ^{1}H NMR spectrum (A), and (B & C) expansion spectrum of compound 6 (hymedesmoside)
Figure S26. ^{13}C NMR spectrums of compound 6 (hymedesmoside)
Figure S27. ^{1}HNMR & ^{13}CNMR data of compound 6 (hymedesmoside)

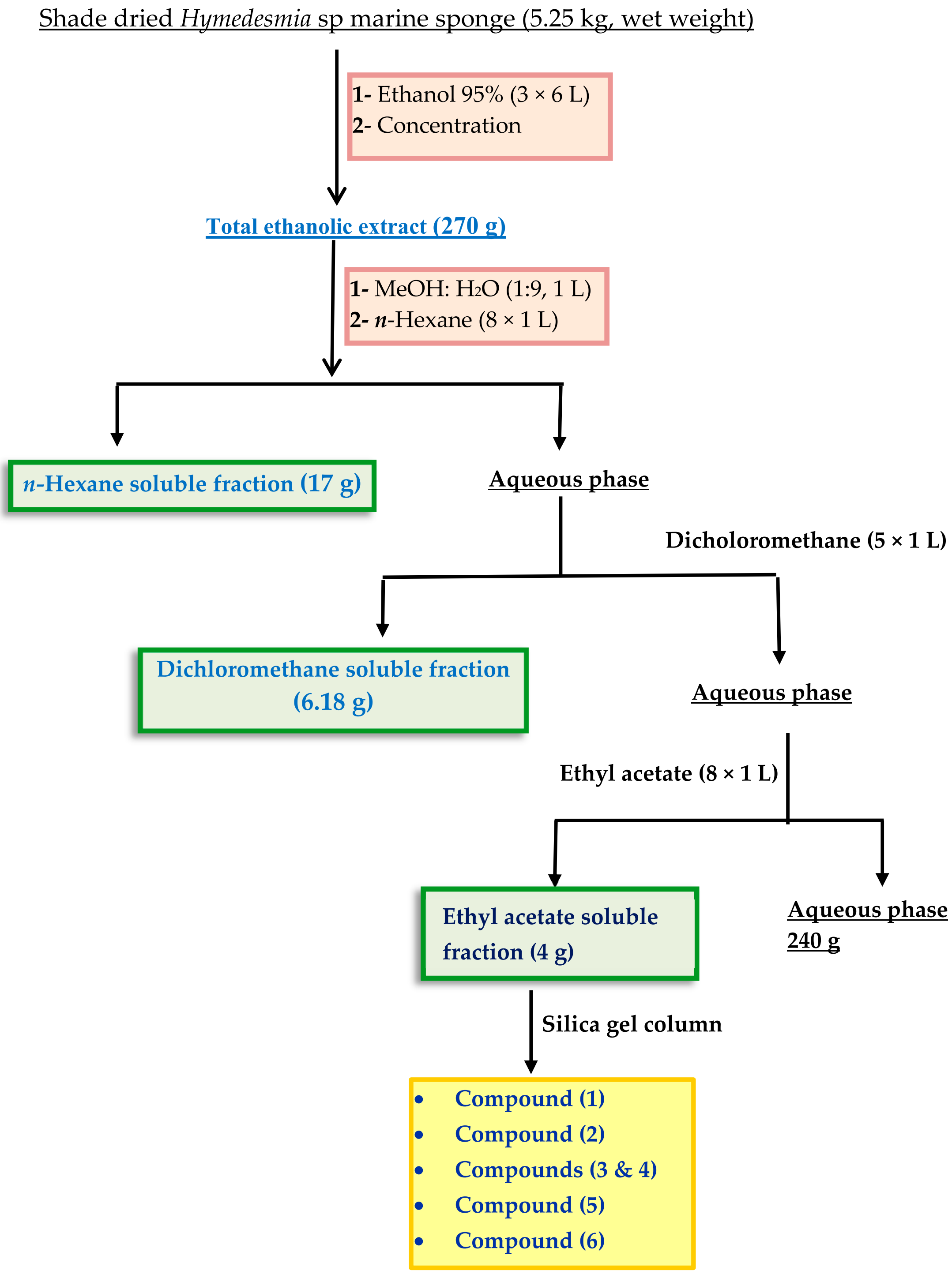

Scheme S1. Extraction and isolation of *Hymedesmia* sp. marine sponge

Table S1. ^{1}H-NMR & ^{13}C-NMR (400 MHz and 100 MHz, DMSO) of compounds 1 (thymine).

No.	δ_H, ppm [mult., *J*(Hz)]	δ_c, ppm (mult.)
1	(NH) 10.80 (br. *s*)	---
2	---	151.5 (C)
3	(NH) 10.80 (br. *s*)	---
4	---	164.9 (C)
5	---	137.7 (CH_3)
6	7.24 (*s*)	107.6 (C)
7	1.72 (*s*, CH_3)	11.8 (CH_3)

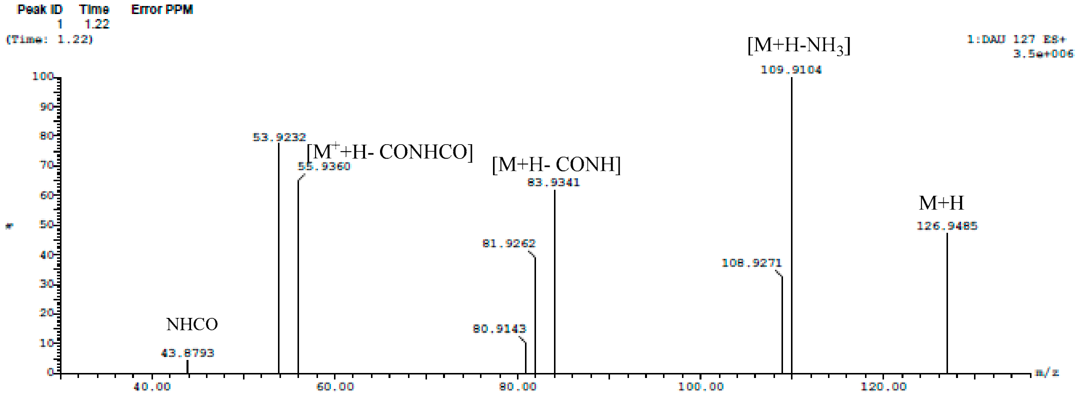

Figure S1. UPLC-ESI-MS/MS chromatogram of compounds 1 (thymine).

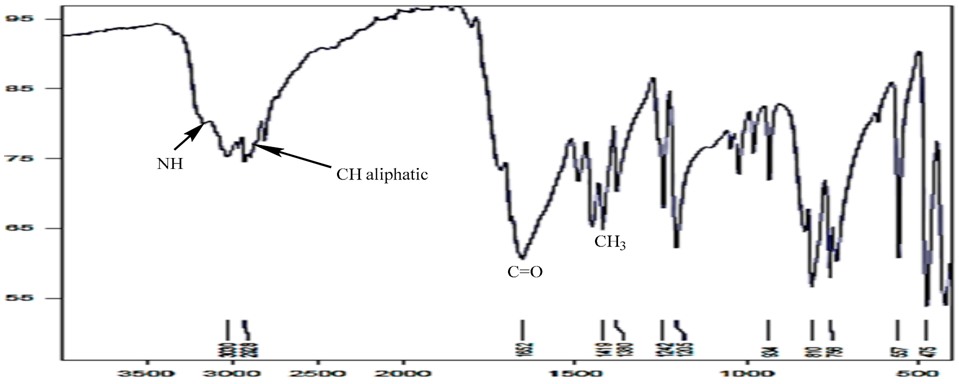

Figure S2. IR spectrum compounds **1** (thymine).

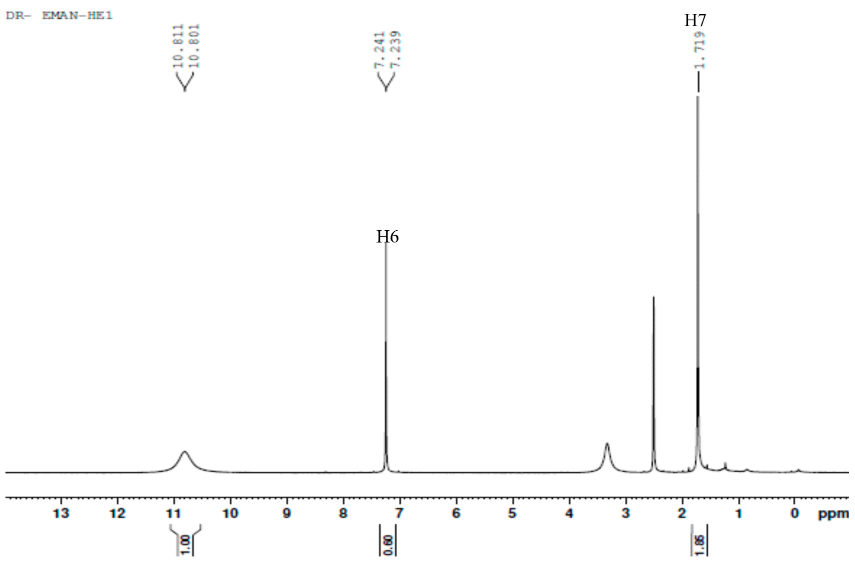

Figure S3. ^{1}H NMR spectrum compounds **1** (thymine).

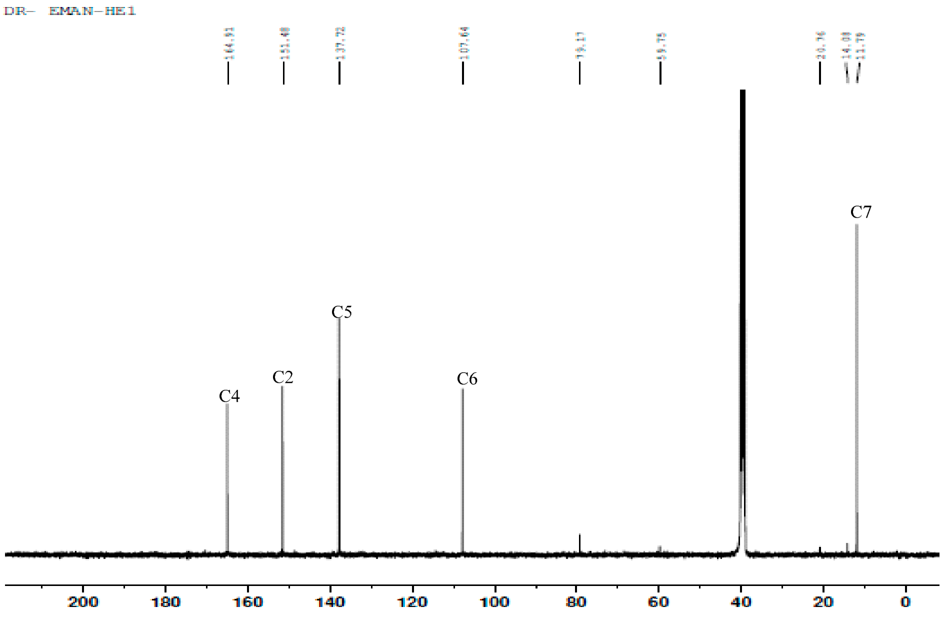

Figure S4. ^{13}C NMR spectrum compounds **1** (thymine).

Table S2. ^{1}H-NMR and ^{13}C-NMR (400 MHz and 100 MHz, DMSO) spectral data for compound **2** (uracil).

Position	δ$_C$ ppm (mult.)	δ$_H$ ppm [mult., *J*(H$_z$)]
1	---	(NH) 10.99 (br. *s*)
2	151.9 (C)	---
3	---	(NH) 10.80 (br. *s*)
4	164.8 (C)	---
5	100.7	5.45 ppm (*d*, *J* = 7.6 Hz)
6	142.7	7.39 ppm (*d*, *J* = 7.6 Hz)

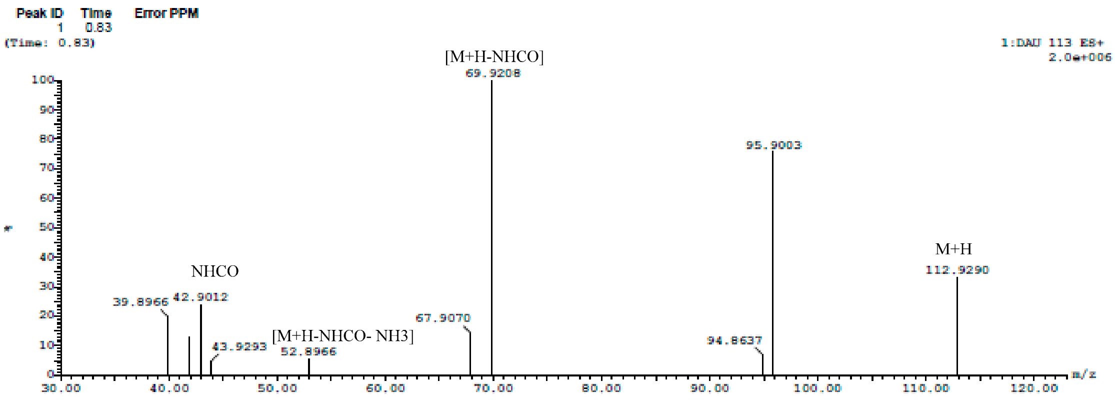

Figure S5. UPLC-ESI-MS/MS chromatogram of compound **2** (uracil).

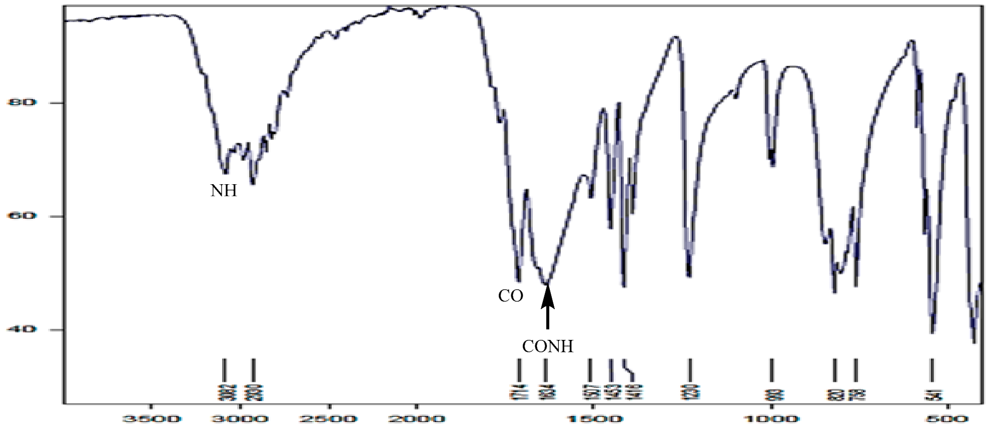

Figure S6. IR spectrum of compound **2** (uracil).

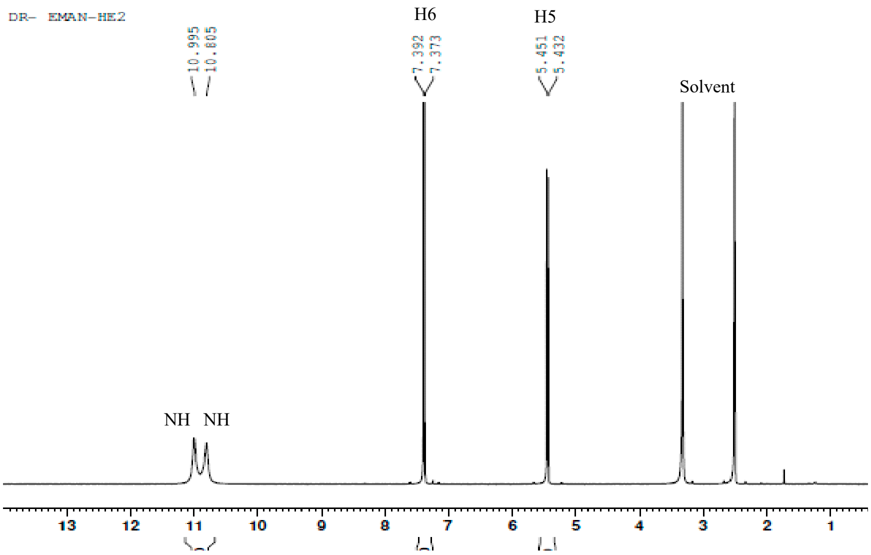

Figure S7. ^{1}HNMR spectrum of compound **2** (uracil).

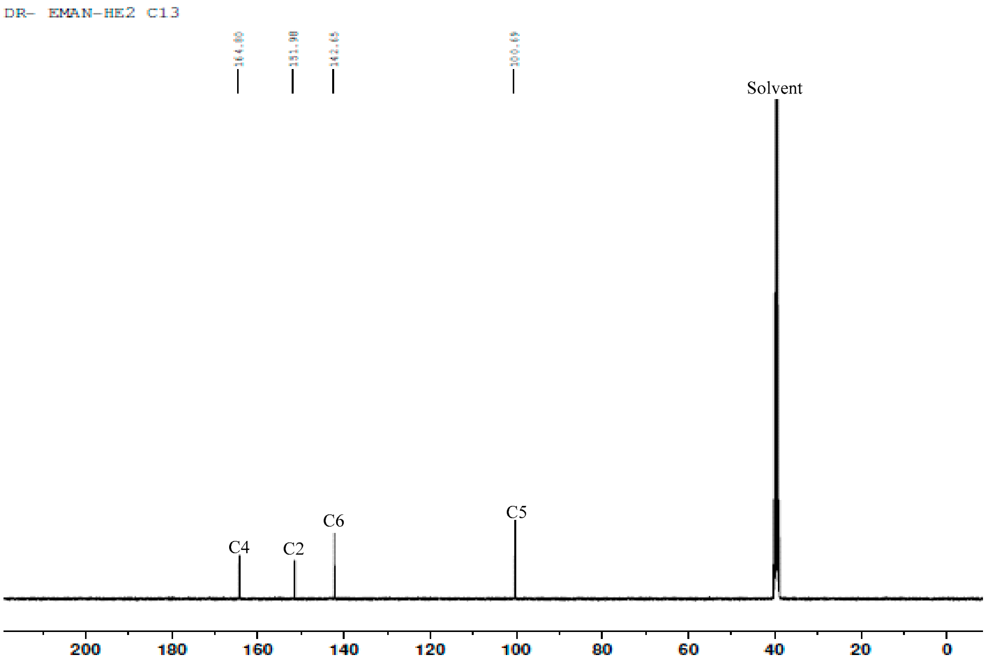

Figure S8. ^{13}C NMR spectrum of compound **2** (uracil).

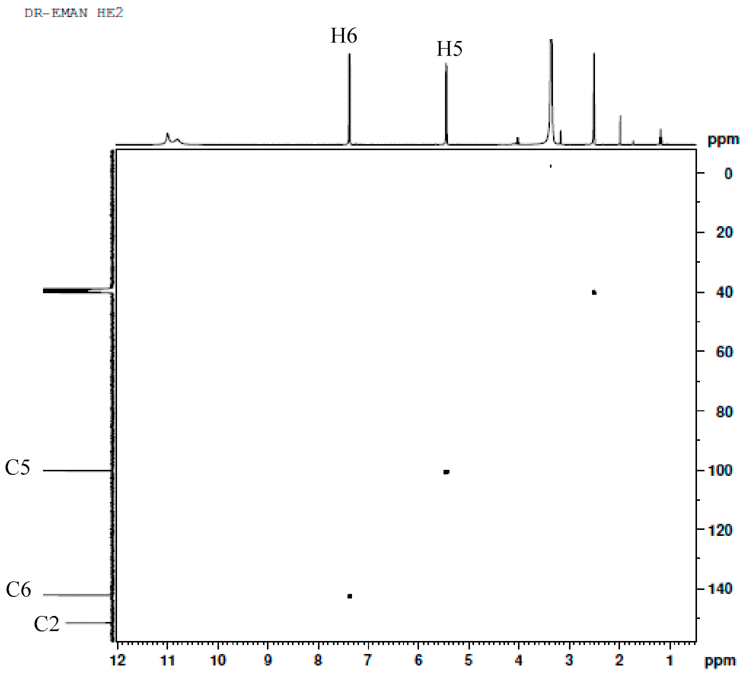

Figure S9. HSQC spectrum of compound **2** (uracil).

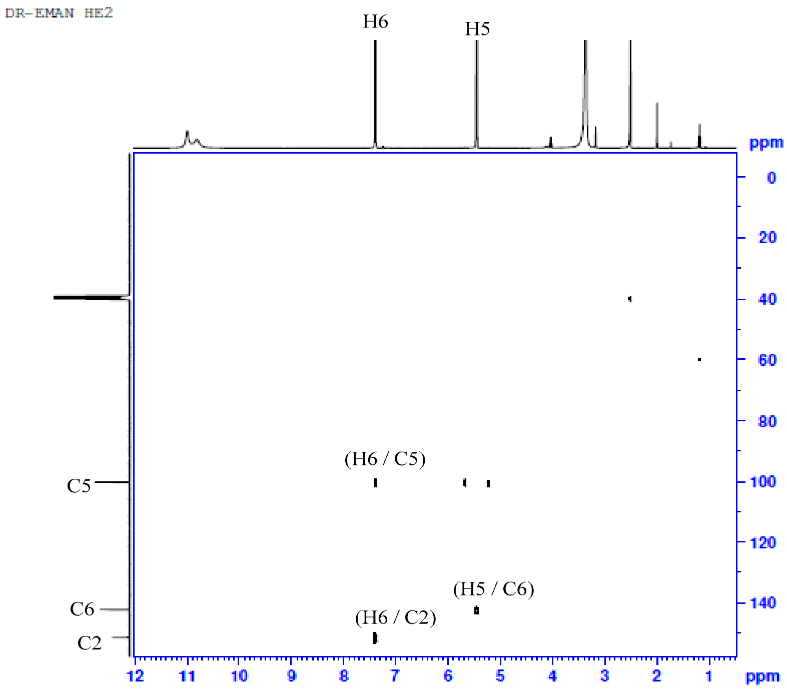

Figure S10. HMBC spectrum of compound **2** (uracil).

Table S3. ^{1}H-NMR and ^{13}C-NMR (400 MHz and 100 MHz, DMSO) spectral data for compounds **3** & **4** (thymidine & uridine).

No.	**Compound 7** (Thymidine) **(DMSO)**		**Compound 8** (Uridine) **(DMSO)**	
	**δ_C , ppm**	**δ_H, ppm [mult., *J*(Hz)]**	**δ_C , ppm**	**δ_H, ppm [mult., *J*(Hz)]**
**1**	--	--	--	--
**2**	150.6	--	150.55	--
**3**	--	11.26 NH	--	11.26 NH
**4**	163.8	--	163.27	--
**5**	136.2	--	101.84	5.65 (d)
**6**	109.5	7.68 (*s*)	140.63	7.80 (d)
**1`**	87.3	6.16	101.84	6.16 (*t*, *J*= 6.4, 7.2 Hz)
**2`**	40.0	2.07	70.52	4.23 (*d*)
**3`**	70.5	4.23	70.52	4.23 (*d*)
**4`**	83.8	4.23	87.48	3.76 (*q*)
**5`**	61.4	3.76	61.41	3.57(*t*)
**Methyl**	12.3	1.77		

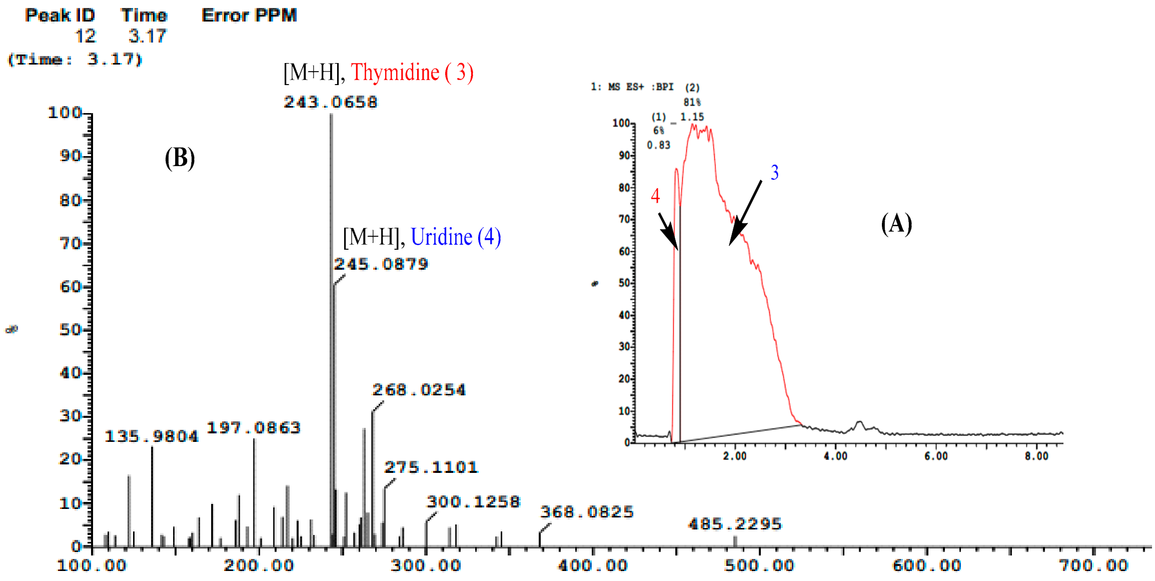

Figure S11. (A): UPLC-ESI-MS chromatogram of compounds **3** & **4** (thymidine & uridine) mixture in positive (+) ionization mode. **(B):** UPLC-ESI-MS/MS chromatogram of compounds **3** & **4** (thymidine & uridine) mixture in positive (+) ionization mode.

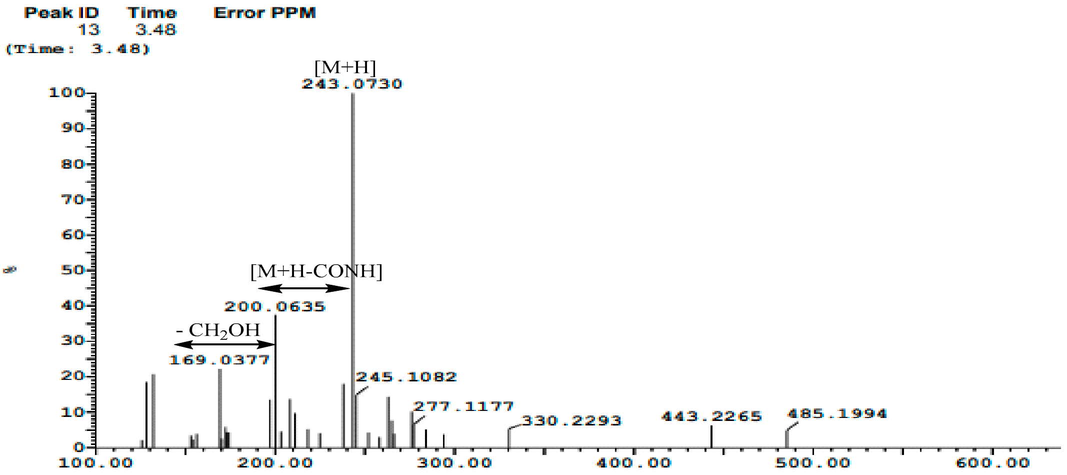

Figure (S12). UPLC-ESI-MS chromatogram of compound **3** thymidine (major, 81%) in positive (+) ionization mode.

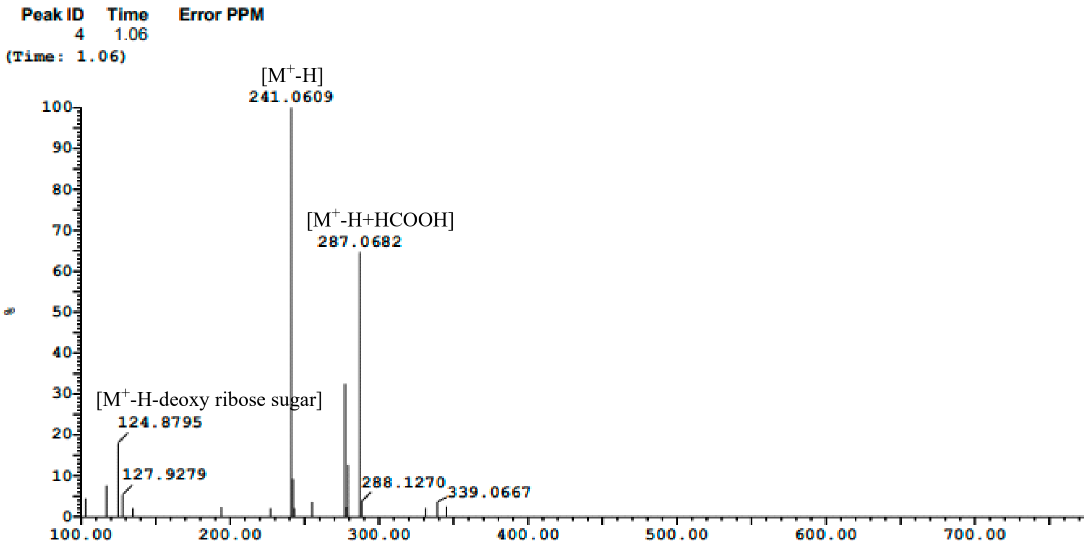

Figure (S13). UPLC-ESI-MS chromatogram of compound **3** thymidine (major, 81%) in negative (-) ionization mode.

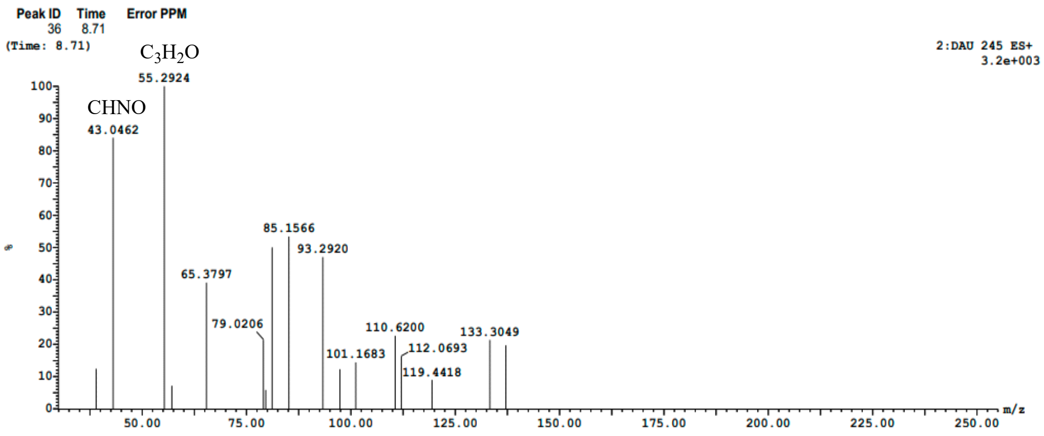

Figure (S14). UPLC-ESI-MS chromatogram of compound **4** uridine (minor, 6%) in negative (+) ionization mode.

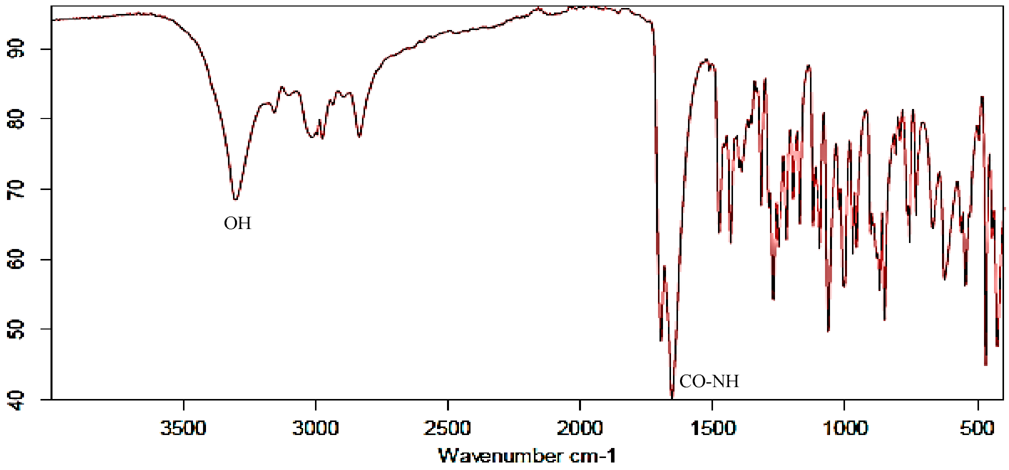

Figure S15. IR spectrum of compounds **3** &**4** (thymidine & uridine).

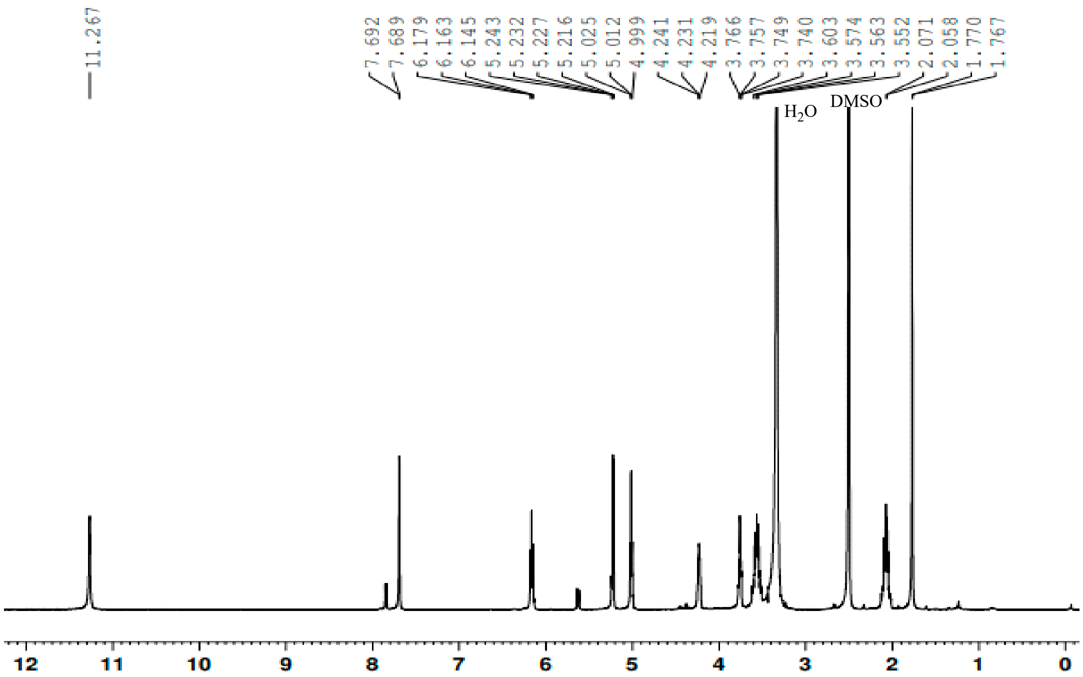

Figure S16. ^{1}HNMR spectrum of compounds **3** & **4** (thymidine & uridine).

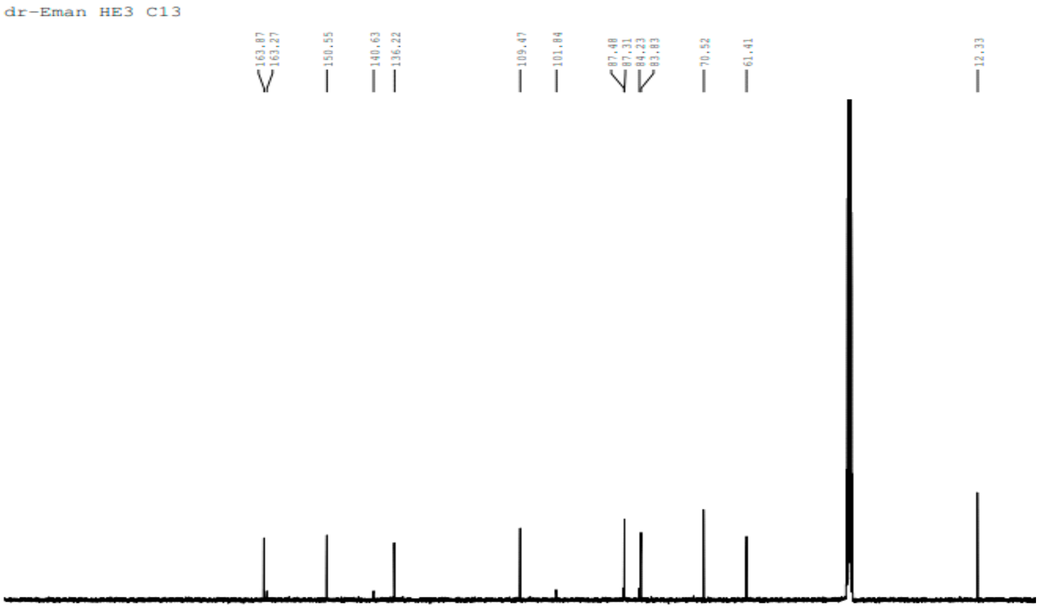

Figure S17. ^{13}CNMR spectrum of compounds **3** & **4** (thymidine & uridine).

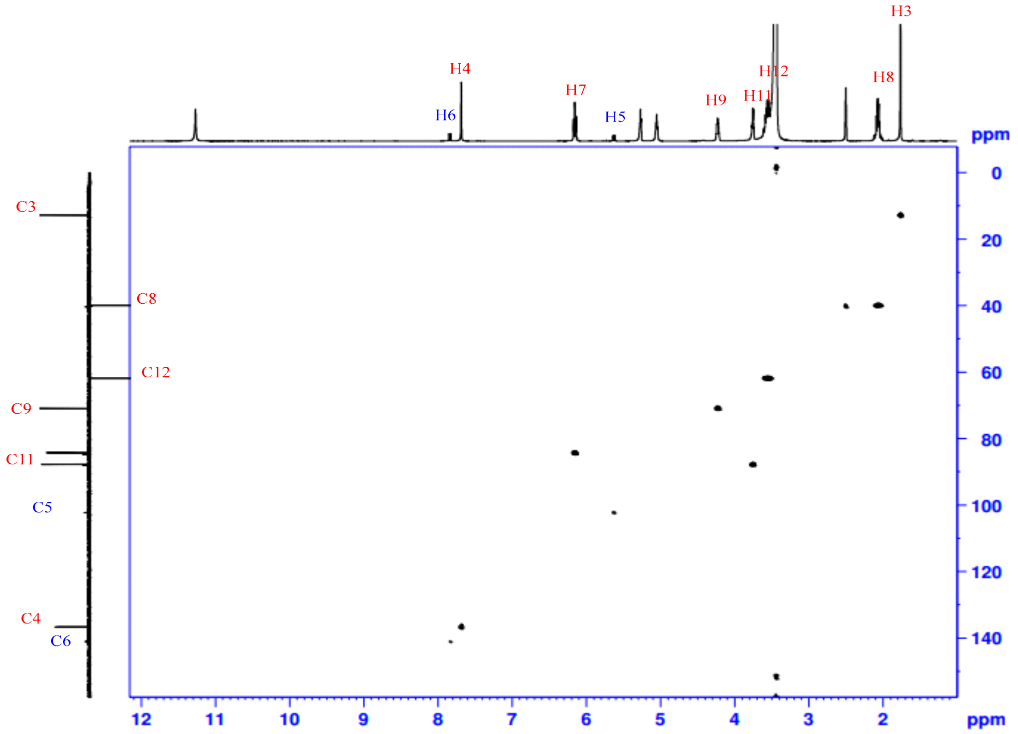

Figure **S18.** HSQC spectrum of compound **3** & **4** (Thymidine & Uridine).

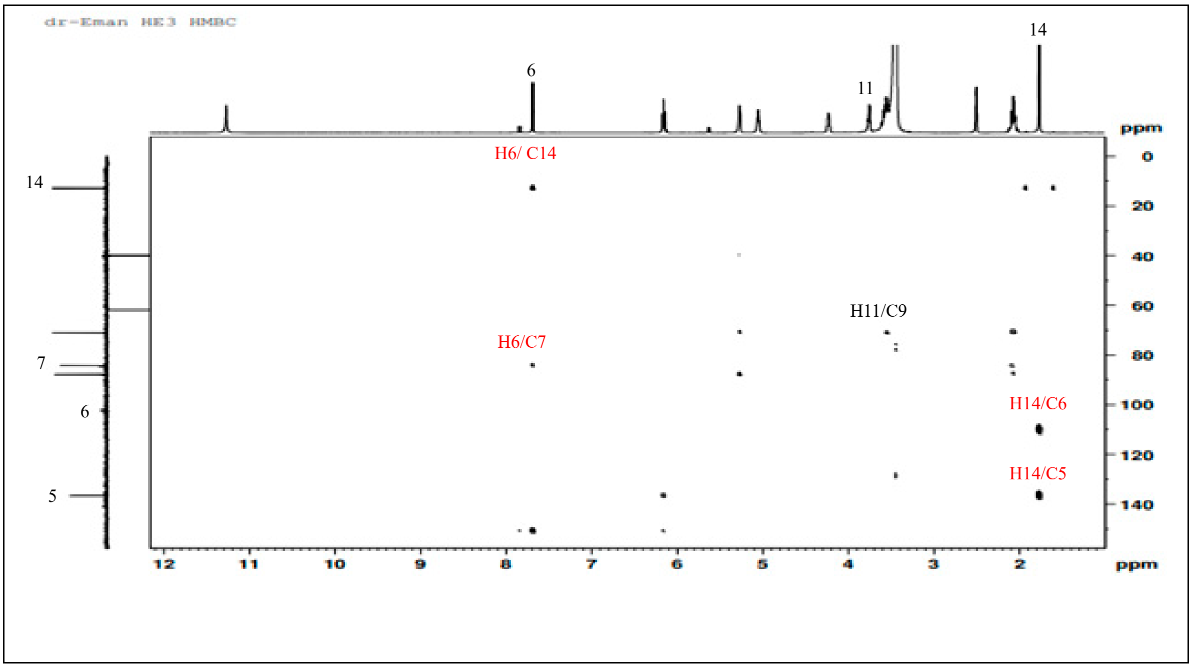

Figure S19. HMBC spectrum of compounds **3** & **4** (thymidine & uridine).

Table S4. ^{1}H-NMR (400 MHz, DMSO) spectral data for compound **5** (adenine).

Position	Atom	δH, ppm [mult., *J*(Hz)]
2	H	8.30 (*s*)
8	H	8.10 (*s*)
9	NH	12.5 (Very broad singlet)
10	NH_2	7.96 (*s*)

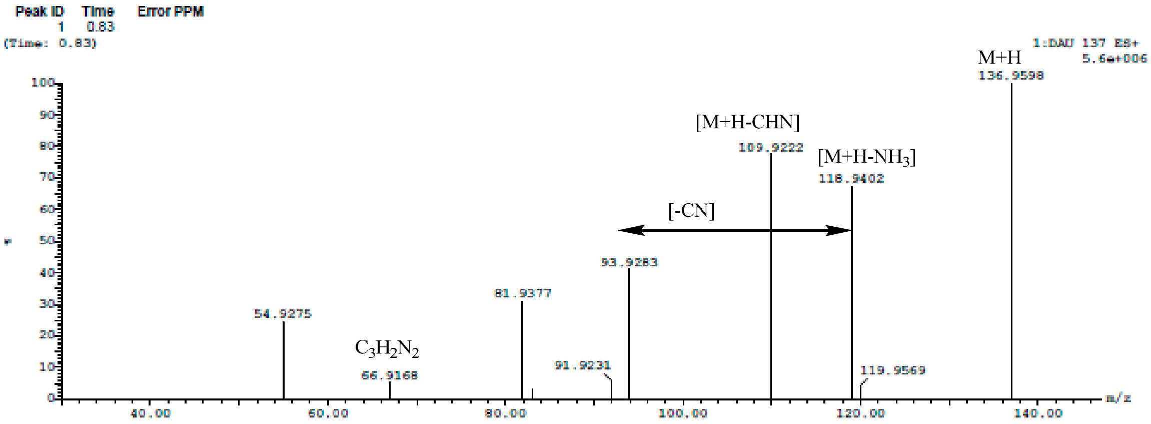

Figure S20. ESI-MS/MS/MS spectrum of compound **5** (adenine).

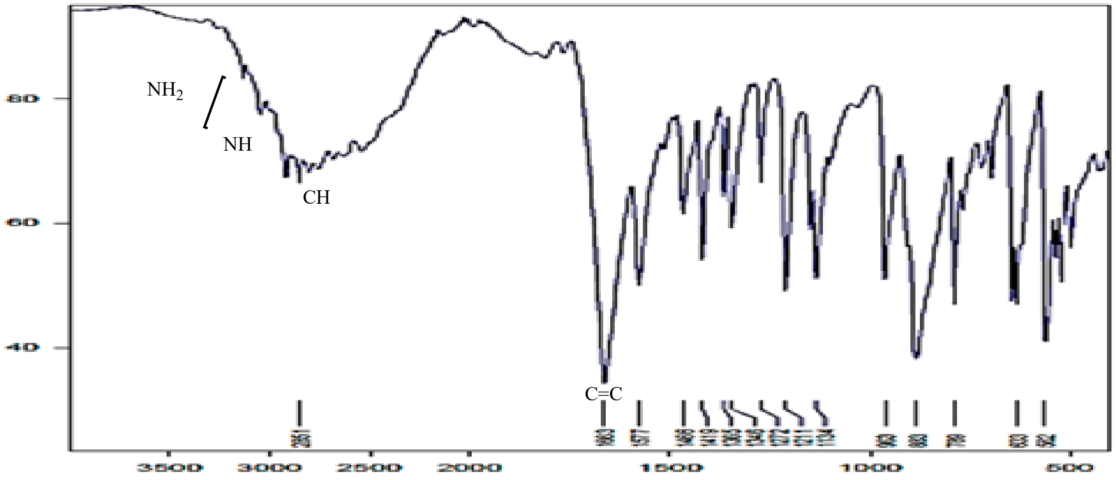

Figure S21. IR spectrum of compound **5** (adenine).

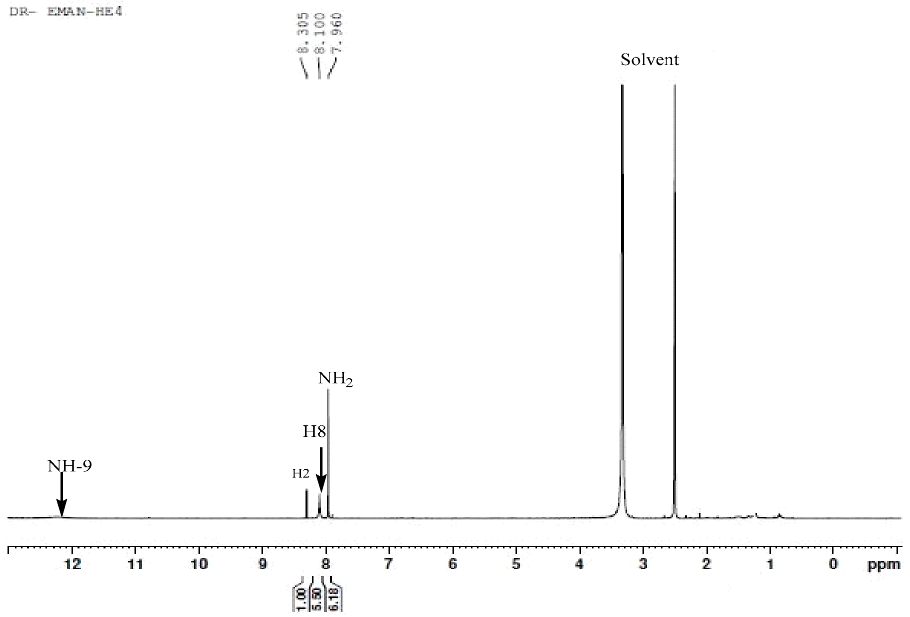

Figure S22. ^{1}HNMR spectrum of compound **5** (adenine).

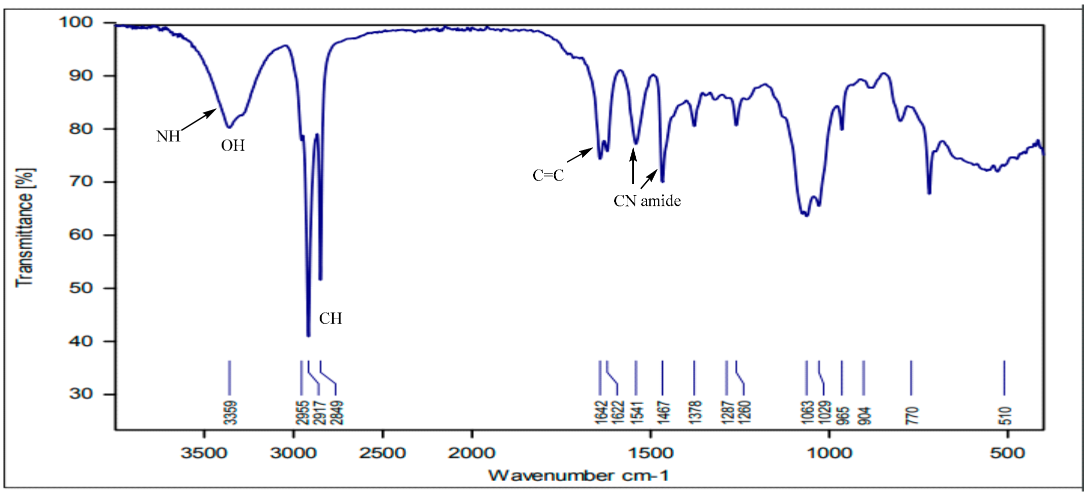

Figure S23. IR spectrum of compound **6** (hymedesmoside).

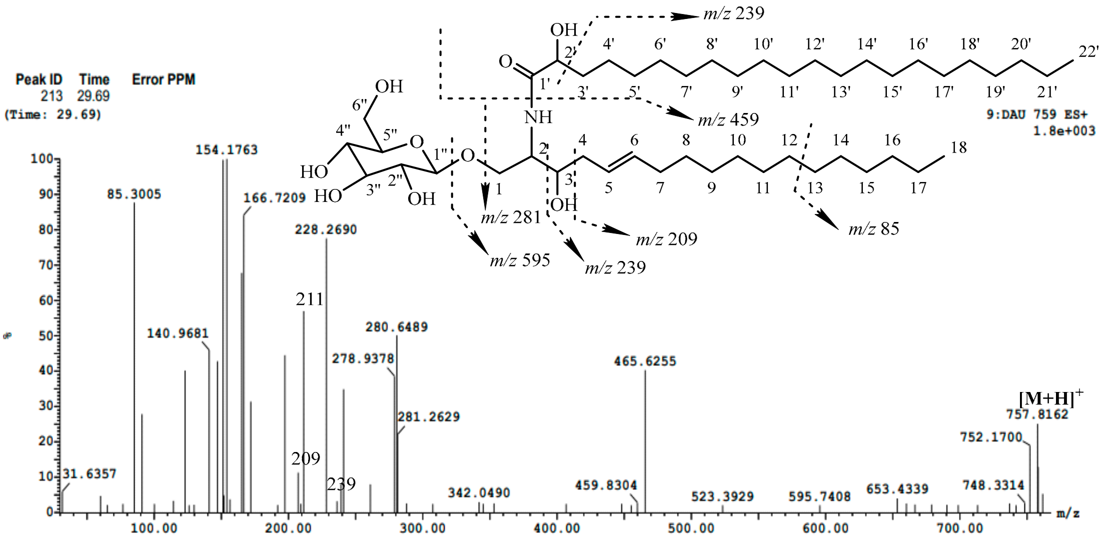

Figure S24. Positive ESI-MS/MS spectrum of compound **6** (hymedesmoside).

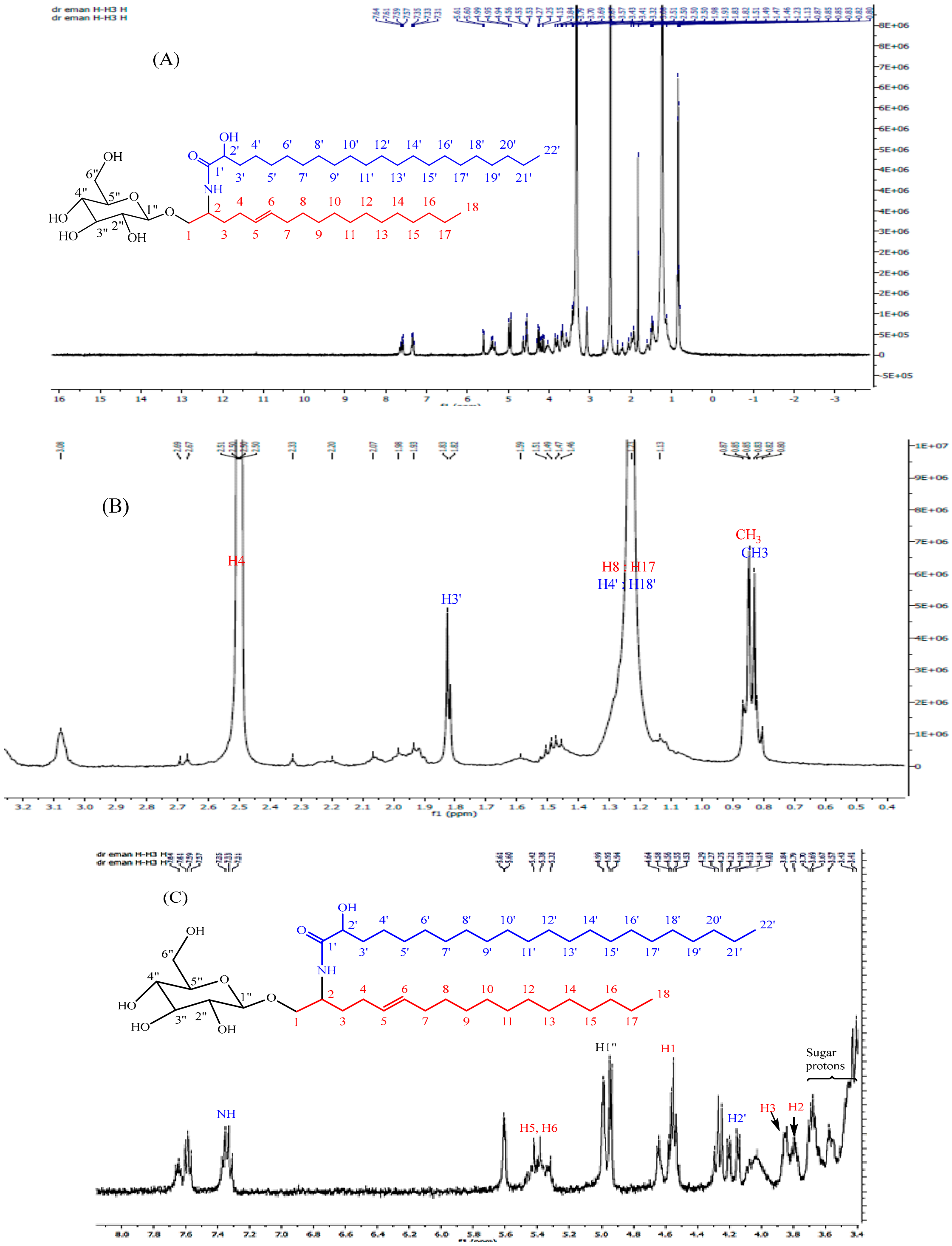

Figure S25. ^{1}H NMR spectrum (A), and (B & C) expansion spectrum of compound **6** (hymedesmoside).

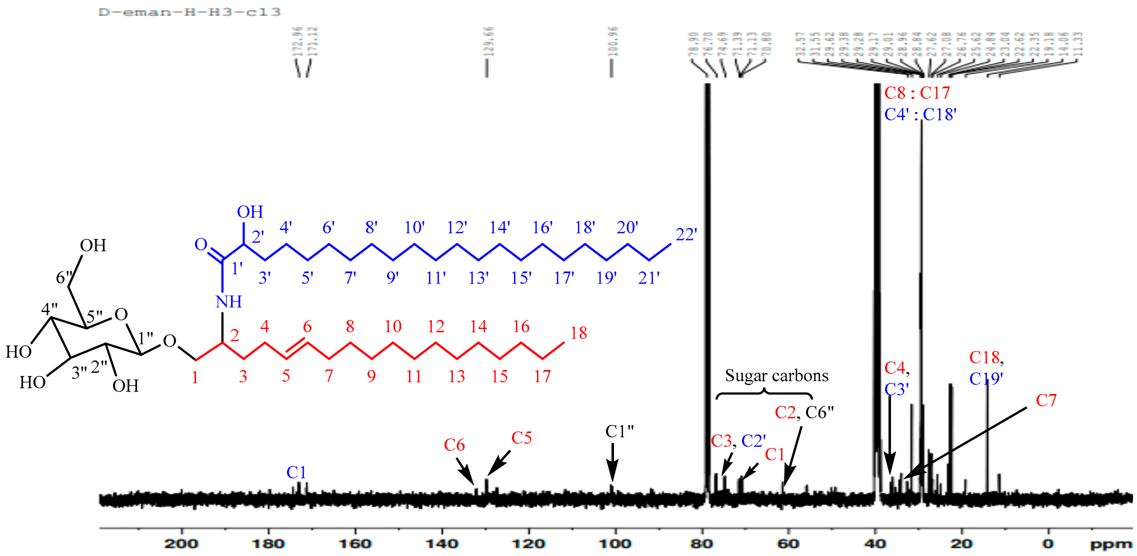

Figure S26. ^{13} C NMR spectrums of compound **6** (hymedesmoside).

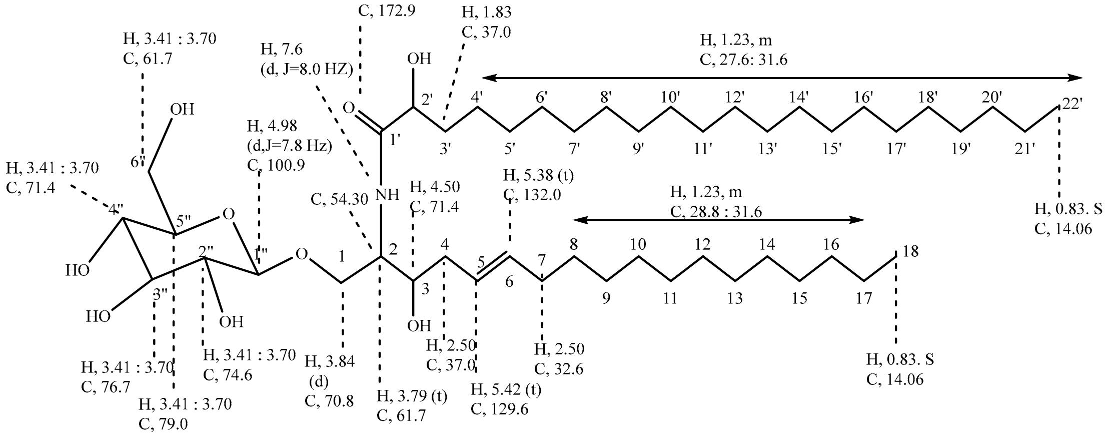

Figure S27. ^{1}HNMR & ^{13}CNMR data of compound **6** (hymedesmoside).
